# Targeting Non-*albicans Candida* Strains and Phytopathogenic *Fusarium* Species with Chitooligosaccharides: Insights into the Antifungal Mechanism

**DOI:** 10.1021/acsomega.5c08432

**Published:** 2025-12-06

**Authors:** Mayara I. G. Azevedo, Nadine M. S. Araujo, Filipe A. Vieira, Milena B. Cherene, Bruno R. S. Queiroz, Daniele O. B. Sousa, Celso S. Nagano, Rômulo F. Carneiro, Valdirene M. Gomes, Rafael G. G. Silva, José E. Monteiro-Júnior, Cosme S. Sousa, Bruno L. Sousa, Thalles B. Grangeiro

**Affiliations:** † Programa de Pós-Graduação em Bioquímica, Departamento de Bioquímica e Biologia Molecular, Centro de Ciências, 426046 Universidade Federal do Ceara, Campus do Pici, Fortaleza, CE 60440-900, Brazil; ‡ Laboratório de Fisiologia e Bioquímica de Microrganismos, Centro de Biociências e Biotecnologia, Universidade Estadual do Norte Fluminense Darcy Ribeiro, Campos dos Goytacazes, RJ 28013-602, Brazil; § Departamento de Engenharia de Pesca, Centro de Ciências Agrárias, Universidade Federal do Ceara, Campus do Pici, Fortaleza, CE 60440-900, Brazil; ∥ Departamento de Biologia, Centro de Ciências, Universidade Federal do Ceara, Campus do Pici, Fortaleza, CE 60440-900, Brazil; ⊥ Faculdade de Filosofia Dom Aureliano Matos, Universidade Estadual do Ceara, Limoeiro do Norte, CE 62930-000, Brazil

## Abstract

The worldwide emergence of resistance to commonly used licensed antifungal drugs, such as azoles and echinocandins, has raised significant concerns for public health and stimulated the search for new antifungal candidates. Chitooligosaccharides (COSs) are water-soluble, biocompatible compounds that have several potential applications in diverse fields, such as human health (e.g., as antimicrobial agents or immune system modulators) and agriculture (e.g., as plant growth promoters or biopesticides). Several *Candida* species, such as *Candida albicans*, affect human health by causing invasive infections such as candidemia. Similarly, *Fusarium* species are economically important phytopathogenic fungi that lead to significant crop losses in wheat, maize, and various other agricultural plants. This work aimed to evaluate the antimycotic activity of low-molecular-weight COSs against *Candida* strains and phytopathogenic fungi. The COS mixture was obtained via hydrolysis of commercial chitosan via the bacterial GH46 chitosanase *Cv*Csn46 from *Chromobacterium violaceum*. Mass spectrometry (ESI-MS) analysis of the enzymatically prepared COS mixture revealed the predominant presence of the monosaccharide β-d-glucosamine (GlcN) and its oligomers (GlcN_2–4_). In silico protein–ligand complexes between a GlcN tetrasaccharide and both the open (apo-form) and closed (substrate-bound form) conformation tridimensional models of *Cv*Csn46 were generated, providing insights into the possible mechanism responsible for the formation of GlcN and GlcN_2–3_ through the hydrolysis of GlcN_4_ and other GlcN oligomers. Additionally, FTIR spectroscopy analysis confirmed the characteristic amine and hydroxyl group signals in the enzymatically produced COS samples. Broth microdilution and time-to-kill assays revealed that the COS samples inhibited the growth of *Candida krusei* (*Pichia kudriavzevii*), *C. parapsilosis* and *C. tropicalis* *in vitro*, exhibiting both fungistatic (minimum inhibitory concentration range: 78–312 μg/mL) and fungicidal (minimum lethal concentration range: 156–625 μg/mL) activities. Among the filamentous fungi tested, COSs significantly (*p* < 0.05) inhibited the mycelial growth of *F. oxysporum* (IC_50_ = 298 μg/mL) and *F. solani* (IC_50_ = 316.5 μg/mL) *in vitro*. Fluorescence and scanning electron microscopy revealed that treatment with COSs caused cell membrane disruption and permeabilization, increased the production of reactive oxygen species (ROS), and led to morphological alterations such as cell shrinkage and surface deformation, ultimately resulting in the death of susceptible strains of *Candida* and *Fusarium*. The effects of ionic strength and pH on the antifungal action of COSs revealed that both ionic and nonionic interactions play a role in their antimicrobial activity. Collectively, the results revealed that COSs act primarily on the cell membrane of susceptible fungi and are promising antifungal drug candidates.

## Graphical abstract

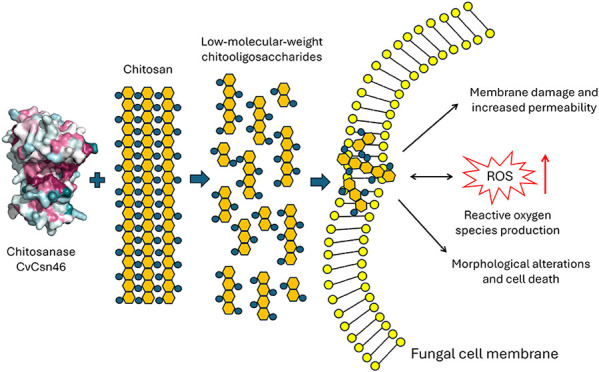

## 1 Introduction

Invasive fungal infections affect more than 6.5 million people and cause more than 3.8 million deaths annually, representing a significant global healthcare problem and economic burden.^1^ Many fungal pathogens have shown increasing resistance to all classes of licensed antifungal drugs, whereas new drug-resistant fungal strains are constantly emerging, making infections even more challenging to treat and increasing the threat to public health.^2,3^ Among the fungal species that cause invasive infections, *Candida*, *Aspergillus*, *Cryptococcus* and *Pneumocystis* are the most clinically relevant.^4^ At least 15 *Candida* species cause human diseases, but more than 95% of invasive infections are caused by only 6 species: *Candida albicans*, *C. glabrata*, *C. tropicalis*, *C. parapsilosis*, *C. krusei* (*Pichia kudriavzevii*) and, more recently, *C. auris*.^5^ *Candida albicans* is the most common cause of candidaemia and invasive candidiasis, but its prevalence has changed in recent years, with a growing number of infections caused by non-*albicans* species (NACs) due to their increasing resistance to antifungals. Compared with other *Candida species*, *Candida krusei* presents intrinsic resistance to fluconazole, and infections with this fungus result in the lowest 90-day survival rate. Furthermore, *C. parapsilosis* is the second to third most isolated *Candida* species from intensive care unit patients, accounting for approximately 80% of newborn infections caused by NACs.^6,7^

In addition to the threat that invasive fungal infections pose to human health, fungal pathogens that infect agricultural crops constitute a significant risk to global food security. Worldwide, farmers lose 10–23% of their crops to fungal infections each year, and an additional 10–20% are lost postharvest.^8,9^ Species of *Fusarium* are among the most economically important phytopathogenic fungi, causing vascular wilt diseases in a large range of crops, including bananas, cotton, barley, maize, wheat, melon, tomato and watermelon. In susceptible varieties of certain crops, *Fusarium* wilt and other diseases caused by *Fusarium* spp. may result in yield losses of up to 100%. Moreover, many *Fusarium* species are mycotoxin producers and opportunistic human pathogens, further threatening human and livestock health.^10,11^

Chitosan is a cationic linear heteropolymer composed of randomly distributed units of 2-acetamido-2-deoxy-β-d-glucopyranose (GlcNAc) and 2-amino-2-deoxy-β-d-glucopyranose (GlcN), linked by β-1,4-glycosidic bonds. This polymer is a partially deacetylated derivative of chitin, with the number of GlcN units usually being greater than 55% in commercial chitosans.^12^ The annual global production of commercial chitosan is approximately 2000 tons, which are derived mainly from seafood waste, mostly from crabs, shrimp and lobsters.^13^ Owing to its abundance, biocompatibility, nontoxicity, biodegradability and other intrinsic functional properties, chitosan and its derivatives have attracted great scientific and industrial interest, with a broad range of practical applications in many fields, including the food industry, agriculture, pharmacy, textile and paper industries, and medicine.^14^ The broad-spectrum antimicrobial properties of chitosan against Gram-positive bacteria, Gram-negative bacteria and fungi have been reported, indicating its potential applications as an alternative antimicrobial agent in medicine and agriculture.^15,16^ However, the poor solubility of chitosan in aqueous solutions with pH values >6 and its high viscosity are properties that limit its application in several fields.^17^

Chitosan oligosaccharides (COSs), also referred to as chitooligosaccharides and chitosan oligomers, are derivatives of chitosan obtained through chemical, physical or enzymatic degradation and are characterized by a low molecular weight (MW) of less than 3.9 kDa, a low degree of polymerization (DP), ranging from 2 to 20, and a high degree of deacetylation (DD), usually >90%.^18^ Unlike chitosan, COSs are highly soluble at neutral pH, have low viscosity, are easily biodegraded, are biocompatible, and have low toxicity. These properties make them suitable for applications in the food and pharmaceutical industries, agriculture, and human and veterinary medicine. The many potential applications of COS are based on their biological effects, including antimicrobial, antioxidant and anti-inflammatory activities, as well as their ability to act as elicitors of plant defense responses against pathogens and pests.^19,20^

The generation of COS through enzymatic hydrolysis has several advantages over chemical and physical depolymerization methods, including higher selectivity and lower energy and environmental costs, and is considered a green technology and a nonhazardous process.^21^ The ability to produce COS mixtures that are well-defined in terms of degree of polymerization or molecular weight, fraction of *N*-acetylated residues (F_A_), degree of *N*-acetylation (DA)/*N*-deacetylation (DD), and pattern of *N*-acetylation (P_A_) or sequence is highly important for better understanding the relationships between their bioactivities, such as antifungal effects, and their structural parameters.^22^ Among the physicochemical properties of COS, the molecular weight, which is correlated with the degree of polymerization, as well as the degree of *N*-deacetylation, are considered the key structural features that influence COS antifungal activity, but other factors also play a role in this process, including their concentration, the pH and ionic strength of the medium, and the target fungal species.^23^

Although many studies have highlighted the antifungal activities of chitosan and chitosan oligomers produced by chemical hydrolysis, there are few reports on the antifungal action of well-defined mixtures of chitooligosaccharides or even pure COS preparations produced by enzymatic hydrolysis that exhibit antimicrobial action against *Candida* and *Fusarium* species.^23–27^ Furthermore, previous investigations have reported contradictory results on the relationship between the COS MW and antifungal activity. While some studies have reported a positive correlation between the COS MW and antifungal action, with higher-MW COSs exhibiting higher antifungal activity, other studies have reported an inverse correlation.^28–31^ These discrepancies in the correlation data between antifungal activity and COS molecular weight have been attributed to several factors, including the use of ill-defined COS samples, differences in the cell wall composition of the tested pathogens, and experimental conditions such as pH, concentration and the method used in sample preparation.^32,33^ Thus, using well-defined COS products with specified MWs, DPs, and DDs is essential to address this issue. The specific objective of this work was to evaluate the inhibitory effects of a well-defined COS mixture produced via the enzymatic hydrolysis of commercial chitosan against *Candida* strains and filamentous fungi, which are involved in human and plant infections, and to elucidate the inhibition mechanism. The antifungal action of the low-molecular-weight COS mixture containing GlcN_2–4_ and free GlcN against *Candida* (*C. krusei* and *C. parapsilosis*) and *Fusarium* (*F. oxysporum* and *F. solani*) indicated a mechanism involving permeabilization of the cell membrane, induction of ROS production and morphological alterations, leading to cell death. Therefore, low-molecular-weight COSs are promising antifungal agents with the potential to be exploited as alternative molecular tools to fight human and plant fungal pathogens.

## 2 Materials and Methods

### 2.1 Chitosan, Fungal Cells, Culture Media and Chemical Reagents

Commercial chitosan powder (DD as provided by the manufacturer = 90%) was purchased from Êxodo Científica (Sumaré, SP, Brazil). The yeast strains used in the growth inhibition tests were *C*. *albicans* (ATCC 10231, ATCC 44858, ATCC 64129, ATCC 90028 and ATCC 90029), *C. krusei* (*P. kudriavzevii*) (ATCC 6258), *C. parapsilosis* (ATCC 22019 and ATCC 90018) and *C. tropicalis* (ATCC 750 and ATCC 13803). Isolates of the filamentous fungi *Colletotrichum gloeosporioides*, *C. lindemuthianum*, *C. scovillei*, *Fusarium lateritium*, *F. oxysporum*, *F. solani*, *Mucor circinelloides* and *Penicillium decumbens* were obtained from the Department of Microbiology, Federal University of Pernambuco (Brazil). Culture media were purchased from HiMedia Laboratories Pvt. Ltd. (Mumbai, India). All other reagents were of high purity and analytical grade.

### 2.2 Molecular Weight Determination of Chitosan

The viscosity-average molecular weight (*M*_w_) of chitosan was determined as previously described.^34^ Chitosan was dissolved in acetic acid (0.1 M acetic acid and 0.2 M NaCl, pH 3), and relative viscosity (η) measurements were performed with a Cannon-Frensk capillary viscometer at 30 ± 0.5 °C. The molecular weight was calculated according to the Mark–Houwink–Sakurada (MHS) equation:

$$\left[\eta \right]=KM_{v}^{a}$$

The constants *K* and a were 1.81 × 10^–3^ g/mL and 0.93, respectively.^35^

### 2.3 Production of Chitooligosaccharides by Enzymatic Hydrolysis of Polymeric Chitosan

Chitooligosaccharides were produced via the enzymatic hydrolysis of colloidal chitosan with a recombinant GH46 chitosanase (*Cv*Csn46) from *Chromobacterium violaceum* ATCC 12472, as previously described.^36^ Briefly, chitosan powder was dissolved (1%, m/v) in 50 mM acetic acid, recombinant chitosanase was added to a final concentration of 5 μg/mL, and the mixture was incubated at 50 °C for 24 h under constant orbital agitation (120 rpm). The reaction was quenched in a water bath at boiling temperature for 10 min, cooled to room temperature and centrifuged (10,000*g* for 20 min at 10 °C). The clear supernatant containing the soluble oligomers was freeze-dried and stored at 4 °C until use.

### 2.4 Molecular Mass Profile of COS

The molecular mass profile of the COS preparations was determined via electrospray ionization mass spectrometry (ESI-MS) with a Synapt G1 HDMS Q-ToF mass spectrometer (Waters Co., Milford, MA) coupled to a Waters ultrahigh-performance liquid chromatography (UPLC) unit. Mass spectra acquisition and data collection and processing were performed as described by Azevedo et al.^36^

### 2.5 Fourier Transform Infrared Spectroscopy (FTIR) Analysis

Characterization of the functional groups of commercial chitosan and COS was performed via FTIR analysis with a VERTEX 70v FT-IR spectrometer (Bruker Optik GmbH, Ettlingen, Germany). Infrared spectra were acquired at a spectral resolution of 4 cm^–1^ with 32 scans per spectrum over a wavenumber range of 4000–400 cm^–1^, following the equipment manufacturer’s instructions.

### 2.6 Determination of the Degrees of Acetylation (DA) and Deacetylation (DD) of Chitosan and COS

The degrees of acetylation (DA) and deacetylation (DD) of chitosan and COS were determined via FTIR spectroscopy, according to the method proposed by Brugnerotto et al.,^37^ which uses the absorbances of the bands at 1320 cm^–1^ (C–N stretching of amide III) and 1420 cm^–1^ (angular deformation of CH_2_), expressed by the following relationships:

$$\mathrm{DA}\%=\left(\left(A_{1320}/A_{1420}\right)-0.3822\right)/0.03133$$

DD%=100−DA%

### 2.7 Comparative Protein Structure Modeling and Molecular Docking Calculations

Homology modeling was performed via the program Modeler 10.6.^38^ Two comparative 3D models of *Cv*Csn46 were generated using the X-ray crystallographic structures of the apo-form (PDB ID: 1QGI)^39^ and substrate-bound form (PDB ID: 5HWA)^40^ of the chitosanase CsnMHK1 from *Niallia circulans* as templates. Protein–ligand molecular docking calculations were performed via the software AutoDock Vina 1.1.2.^41^ The search space for the docking calculations was defined by a cubic grid (30 Å × 30 Å × 30 Å), centered on the conserved active site of *Cv*Csn46, and the exhaustiveness was set to 33. The top ten poses, ranked by their predicted binding affinities (expressed in kcal/mol), were examined, and the best results were selected on the basis of a comparative analysis with X-ray crystallographic structures of chitooligosaccharides complexed with chitosanases. The molecular models were visualized using the program PyMOL Molecular Graphics System 3.1.3 (Schrödinger, Inc., New York, NY).

### 2.8 Antifungal Activity Assays of COS against *Candida* Strains

#### 2.8.1 Broth Microdilution Assay

The ability of COS to inhibit the growth of *Candida* strains was evaluated through the broth microdilution method, as previously described.^42^ Serial 2-fold dilutions of COS samples were performed in Sabouraud dextrose broth in 96-well U bottom microplates, and yeast cells cultivated in the same medium were inoculated into the wells to a final concentration of 1 × 10^3^ colony-forming units (CFU)/mL. The cultures were incubated at 35 °C for 24 h and visually inspected. Wells containing cells inoculated in culture medium without COS and wells containing only culture medium were included as positive and negative controls of yeast growth, respectively. The minimal inhibitory concentration (MIC) was defined as the lowest COS concentration at which there was no visible growth. To determine the minimum lethal concentration (MLC), aliquots (5 μL) from each well with no visible growth were inoculated onto Sabouraud agar plates and incubated at 35 °C for 24 h. The MLC was defined as the lowest COS concentration that killed at least 99.9% of the inoculum. All the assays were performed in triplicate.

#### 2.8.2 Agar Diffusion Test

The agar diffusion test was performed as previously described.^42^ A standardized inoculum of each strain, matching the turbidity of a 0.5 McFarland standard, was spread onto Sabouraud agar plates, and droplets of 0.2% agarose gel containing COS at a 0.5% (m/v) concentration were loaded onto the medium. The cultures were incubated at 35 °C for 24 h, and the diameters of the growth inhibition zones were measured using a pachymeter and rounded to the closest millimeter. Droplets of 0.2% agarose gel without COS and droplets of ketoconazole-based antifungal cream were used as controls. All the assays were performed in triplicate.

#### 2.8.3 Time–Kill Kinetics Assay

Time–kill curve assays were performed according to the method described by Klepser et al.,^43^ with modifications. Briefly, suspensions of *Candida* cells (2.5 × 10^3^ to 3 × 10^3^ CFU/mL) were treated with COS at different concentrations (1/2 × MIC, MIC and 2 × MIC), and the cultures were incubated at 35 °C for 24 h. Aliquots from each culture were removed at various time intervals (at 0, 2, 4, 6, 8, 10, 12, and 24 h posttreatment), inoculated onto Sabouraud dextrose agar, incubated at 35 °C for 24 h, and the CFU/mL values were calculated. All the assays were performed in triplicate.

#### 2.8.4 Antibiofilm Effects of COS

The antibiofilm activity was determined through a crystal violet-based assay performed in polystyrene flat-bottom 96-well microtiter plates, as previously described.^44^ To assess the effect of COS on biofilm formation, yeast cell suspensions (1 × 10^6^ CFU/mL) were incubated (37 °C, 48 h, 75 rpm) with COS (1/2 × MIC, MIC and 2 × MIC), the culture supernatant was removed, and the biofilm formed was stained with 0.1% (m/v) crystal violet for 15 min. Excess stain was washed with 0.15 M NaCl, 95% (v/v) ethanol was added to solubilize the crystal violet bound to the cells, the absorbances at 595 nm were measured, and the percentages of biofilm formation were calculated.

To assess the ability of COS to promote the disorganization of a preformed biofilm, yeast cell suspensions were incubated at 37 °C for 24 h, the culture supernatant was removed, and Sabouraud broth and COS (at the same concentrations described above) were added to the preformed biofilm, which was incubated again at 37 °C for 24 h. The supernatant was discarded, the remaining biofilm was stained with 0.1% (m/v) crystal violet, and its biomass was quantified by measuring the absorbance at 595 nm.

### 2.9 Effects of COS on Cell Membrane Integrity and Assessment of Cell Death

The assays used to investigate the effects of COS on *Candida* cell membrane integrity were performed as previously described,^44^ with minor adaptations. Aliquots (1 mL) of yeast cell suspensions (1 × 10^6^ CFU/mL) were incubated with COS (MIC), water (negative control) or 50% (v/v) ethanol (positive control) at 35 °C for 1 h. The cells were subsequently centrifuged (5000*g*, 10 min, 4 °C), resuspended in 0.15 M NaCl (1 mL) and incubated with 1 mM propidium iodide (PI) for 10 min at 35 °C. The cells were then visualized under a fluorescence microscope (Olympus BX60 microscope; excitation wavelength, 490 nm; emission wavelength, 520 nm).

To estimate the percentage of cell death induced by COS, the cells (1 × 10^6^ CFU/mL) were treated with COS (MIC) at 35 °C for 1 h, centrifuged (5000*g* for 10 min at 4 °C), resuspended in 2 mL of 0.15 M NaCl, incubated with 1 μM PI for 10 min and analyzed via flow cytometry via a 535 nm laser line and a 617 nm filter for PI. Flow cytometric detection was performed with a Partec GmbH flow cytometer (Münster, Germany). Cells treated with water or 50% (v/v) ethanol were used as negative and positive controls for cell death, respectively.

### 2.10 Detection of Reactive Oxygen Species (ROS) Generated after Treatment with COS

The procedure described by Souza et al.^44^ was used to detect the ROS generated by *Candida* cells after COS treatment, with slight modifications. Yeast cell suspensions (1 × 10^6^ CFU/mL) were incubated with COS (MIC) or water (negative control) for 1 h at 35 °C. The cell suspensions incubated with 10% (v/v) H_2_O_2_ (35 °C, 15 min) were used as positive controls. The treated cells were subsequently centrifuged (5000*g*, 10 min, 4 °C) and incubated with 10 μM 2′,7′-dichlorofluorescein diacetate (DCFH2-DA) for 30 min at 35 °C. The cells were washed twice with 0.15 M NaCl and visualized under a fluorescence microscope (Olympus BX60 microscope; excitation wavelength, 490 nm; emission wavelength, 520 nm).

### 2.11 Growth Inhibition of Filamentous Fungi

The effect of COS on the mycelial growth of phytopathogenic fungi was determined via the quantitative microtiter plate assay described by Broekaert et al.^45^ The spore suspensions (1 × 10^4^ cells/mL) were treated with COS (7.8 to 1000 μg/mL) for 24 or 48 h at 30 °C, and mycelial growth was quantified by measuring the absorbance at 620 nm.

### 2.12 Effects of COS on the Cell Membrane Integrity and ROS Production of Filamentous Fungi

The SYTOX Green uptake assay^46^ was used to evaluate membrane permeabilization in filamentous fungi treated with COS (IC_50_). The hyphal suspensions were treated with COS (30 °C, 24 h), incubated with 0.2 μM SYTOX Green (25 °C, 10 min) and visualized under a fluorescence microscope (Zeiss AxioVision microscope; excitation wavelength, 450–490 nm; emission wavelength, 500 nm). To detect the intracellular production of ROS, COS-treated hyphal suspensions (IC_50_, 30 min, 24 h) were incubated with 50 μM DCFH2-DA (25 °C, 30 min) and visualized under a fluorescence microscope, as described above.

### 2.13 Scanning Electron Microscopy (SEM) Analysis

The preparation of the samples for examination via SEM was performed as previously described.^47^ Briefly, the cells were washed three times with 0.15 M NaCl and fixed for 16 h with 2.5% glutaraldehyde (prepared in 0.15 M sodium phosphate buffer, pH 7.2). Fixed cells were washed with PBS (0.15 M sodium phosphate buffer, pH 7.2), fixed again with 10% osmium tetroxide, and treated with increasing concentrations of ethanol (0, 30, 50, 70 and 100%). The cells were then dried with hexamethyldisilazane (HMDS), metalized with gold and observed under a Quanta FEG-450 scanning electron microscope instrument (FEI Company, Hillsboro) equipped with a low-energy detector (Everhart-Thornley detector).

### 2.14 Statistical Analysis

All the experiments were performed in triplicate. The data are presented as the means ± standard deviations (SDs) and were subjected to one-way analysis of variance followed by Dunnettʼs post hoc test. Statistical analyses were performed via the software GraphPad Prism 8.4.3. In all the tests, the significance threshold was *P* = 0.05.

## 3 Results and Discussion

### 3.1 Molecular Mass Profile of COS Produced by *Cv*Csn46 and Its Mechanism of Chitosan Hydrolysis

The COS samples used in the antifungal assays were produced via the enzymatic hydrolysis of colloidal chitosan with the chitosanase *Cv*Csn46. The viscosity-average *M*_w_ of the parent polymer was determined to be approximately 157 kDa. The hydrolysates of chitosan obtained after 24 h of incubation at 50 °C contained a mixture of low-molecular-weight chitooligomers with DP values ranging from 1 to 7, as revealed by ESI–MS analysis (Figures S1 and S2). The most intense ion clusters were observed at *m*/*z* values of 180 [(GlcN)_1_], 341 [(GlcN)_2_], 502 [(GlcN)_3_] and 663 [(GlcN)_4_]. Other major ions were observed at *m*/*z* 162 and 144, which correspond to the neutral loss of a H_2_O molecule from the reducing end of (GlcN)_1_ (*m*/*z* 162) and the loss of another H_2_O molecule from the dehydrated monosaccharide (*m*/*z* 144). Likewise, the ions at *m*/*z* 323 and 306 originated from the sequential loss of H_2_O and OH from (GlcN)_2_, respectively (Figure S1A). In addition, cluster ions with very low relative intensities were detected at *m*/*z* values ranging from 842 [(GlcN_5_)] to 1164 [(GlcN)_7_] (Figure S1B). The mass profile of the products released from the hydrolysis of chitosan by *Cv*Csn46, with a predominance of (GlcN)_1–4_, is similar to that observed for the chitosanase CsnMHK1 from *N*. *circulans* MH-K1,^48^ a GH46 enzyme that shares 70.53% sequence identity with the catalytic domain of *Cv*Csn46. These two enzymes (*Cv*Csn46 and CsnMHK1), which can also cleave chitosan in an exotype manner, generating the monosaccharide GlcN as well as GlcN oligomers simultaneously, are a few exceptions among all characterized GH46 chitosanases, which have been shown to be mostly exclusively endotype enzymes.^49^

To better understand how the pattern of products released from chitosan degradation by *Cv*Csn46 is produced, molecular models for their open and closed conformations were generated and validated (Figures S3–S5). Both model structures had Rama-*Z* scores ranging from +2 to −2, indicating normal protein backbone geometry.^50^ The calculated composite QMEANDisCo global scores were 0.82 ± 0.05 and 0.84 ± 0.05 for the open and closed conformations, respectively, indicating high confidence in the models’ structural accuracy.^51^ Structural comparisons between the open and closed models of *Cv*Csn46 and the X-ray crystallographic structures of the chitosanases Csn-PD from *Paenibacillus dendritiformis* (apo-form structure; PDB ID: 7XH0; 79.1% sequence identity with *Cv*Csn46) and CsnMHK1 from *N*. *circulans* (substrate-bound structure; PDB ID: 5HWA)^40^ revealed RMSD values of 0.61 and 0.044 Å, respectively, indicating that both models had a correct topology (Figure S6). The RMSD value between the open and closed forms of *Cv*Csn46 was 2.082 Å (Figure S7).

Protein–ligand complexes of a GlcN_4_ oligomer and open and closed conformation models of *Cv*Csn46 were generated via molecular docking calculations (Figure 1), and redocking experiments were performed to validate the docking accuracy (Figure S8). From the alignment with the prototype GH46 chitosanases CsnMHK1 and CsnN174 from *Streptomyces* sp. N174 (24% sequence identity with *Cv*Csn46),^52^ the catalytic residues of *Cv*Csn46 were identified as Glu^138^ (the general acid residue) and Asp^156^ (the general base residue) (Figure S9), which lie on opposite sides of the substrate-binding groove (Figure 1). A third conserved residue, which was identified as Thr^161^ in *Cv*Csn46, is also essential for catalysis, assisting the general base in the activation of a water molecule for nucleophilic attack of the glycosidic linkage.^49^ In the open conformation, docking simulations revealed that GlcN_4_ binds the substrate-binding cleft of *Cv*Csn46 spanning subsites −3 to +1. In this complex, the position of the side chain of Glu^138^ is not favorable for catalysis, as the Oε1 and Oε2 atoms are more than 6 Å apart (6.1 and 7.4 Å, respectively) from the O atom of the scissile glycosidic bond (Figure 1A). In the closed conformation, the upper and lower domains approach one another upon substrate accommodation, making the Oε1 and Oε2 atoms of Glu^138^ closer to the O atom of the scissile glycosidic bond. The side chains of Asp^156^ and Thr^161^ also assume favorable positions for catalysis (Figure 1B,C). In the closed conformation, the chitotetraose ligand bound the substrate-binding cleft of *Cv*Csn46 in two different but equally favorable orientations, one occupying subsites −3 to +1 (average distance of the Oε atoms of Glu^138^ to the scissile glycosidic bond = 4.2 Å) (Figure 1B) and the other spanning subsites −2 to +2 (average distance of the Oε atoms of Glu^138^ to the scissile glycosidic bond = 3.9 Å) (Figure 1C). Therefore, these distinct binding modes can explain how the chitosanase *Cv*Csn46 probably generates GlcN, GlcN_2_ and GlcN_3_ from the hydrolysis of GlcN_4_ and other GlcN_n_ oligomers that are produced from its endotype action on polymeric chitosan chains.

**Figure 1:**
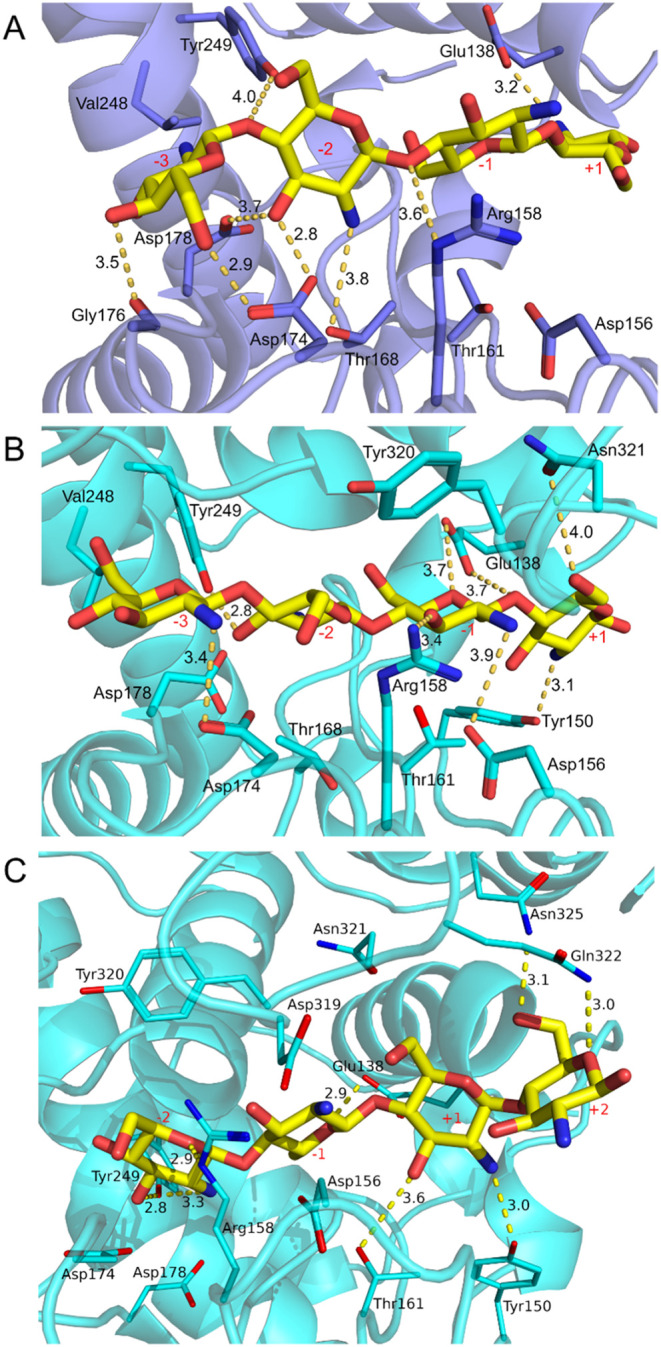
Detailed view of chitotetraose (yellow) docked in the substrate-binding cleft of *Cv*Csn46 three-dimensional models. The ligand was docked in the substrate-binding groove of the open (A) and closed (B and C) forms of *Cv*Csn46. Side chains of residues that interact with the docked ligand through hydrogen bonds (represented as yellow dotted lines) and hydrophobic contacts are shown as sticks (cartoons are colored slate in A and cyan in B and C). N and O atoms are colored blue and red, respectively.

### 3.2 FTIR Analysis of Chitosan and the Antifungal Oligomers Produced by Enzymatic Hydrolysis

To characterize the functional groups of COS and the parent polymer, FTIR spectroscopy analysis was performed in the region of 4000–400 cm^–1^ (Figure S10). The FTIR spectrum of chitosan (Figure S10, blue line) showed the main characteristic absorption peaks in the functional group region (4000–1500 cm^–1^), attributed to the overlapping stretching vibrations of O–H and N–H groups (3500–3100 cm^–1^), the stretching vibrations of C–H bonds (2950–2850 cm^–1^) and the N–H bending vibration of the primary amine (amide II; 1590 cm^–1^), and the characteristic peaks in the fingerprint group region (1200–800 cm^–1^), assigned to stretching vibrations of the C1–O–C4 bridge and stretching vibrations of the pyranose ring C–C and C–O bonds (1150–1000 cm^–1^), and CH bending vibrations out of the plane of the pyranose ring (892 cm^–1^). Bands ascribed to CH_3_ symmetrical deformations (1374 cm^–1^) as well as the bending vibrations of CH_2_ and the primary OH group (6-CH_2_OH) (1418 cm^–1^) were also present. Overall, the FTIR spectrum of the polymeric chitosan agreed with the data reported in previous works.^53,54^ The COS spectrum (Figure S10, red line) was analogous to the spectrum of the precursor polysaccharide but exhibited much more intense and broader absorption peaks. These changes in the IR band areas and band shifts can be attributed to modifications in the environment of the OH and NH_2_ groups due to depolymerization of the chitosan chains, which reduces the crystalline order and increases the degree of conformational disorder, affecting the intra- and intermolecular hydrogen bonding networks.^55^ Conversely, COS spectral bands in the sugar fingerprint region (1200–800 cm^–1^), assigned to stretching vibrations of the C1–O–C4 moiety of the glycosidic linkage and stretching vibrations of the pyranose ring C–C and C–O bonds, had the same pattern and were superimposable with the sugar fingerprint bands of the parent chitosan. Therefore, the fundamental saccharide structural characteristics of the parent polysaccharide are conserved in the COS samples obtained via enzymatic hydrolysis. The DD values for the parent chitosan and the COS sample produced via enzymatic hydrolysis, as determined by FTIR, were approximately 80 and 92%, respectively.

### 3.3 *In Vitro* Antifungal Activity of COS Against Planktonic *Candida* Strains

The antimicrobial action of chitosan and its derivatives has been largely attributed to ionic interactions between the cationic groups of the poly­(oligo) saccharide and anionic components of the microorganism cell surface.^23,56^ As the p*K*_a_ values of chitosan and its chitooligomers vary from 6.0 to 6.5,^57,58^ the anti-*Candida* activity of the COS preparations produced by enzymatic hydrolysis was assessed by cultivating the cells in acidic media (Sabouraud broth, pH 5.5). Under these conditions, COS inhibited the *in vitro* growth of *C. albicans*, *C. krusei*, *C. parapsilosis* and *C. tropicalis* and exhibited both fungistatic and fungicidal activities (Table 1). MIC values ranged from 78 μg/mL (*C. tropicalis* ATCC 750) to 1250 μg/mL (*C. albicans* strains ATCC 64129 and ATCC 90028). COS also had fungicidal effects on 6 strains, with MLC values ranging from 156 μg/mL (*C. tropicalis* strains ATCC 750 and ATCC 13803) to 625 μg/mL (*C. parapsilosis* ATCC 90018). Therefore, chitosan oligomers are generally much more effective as fungistatic and fungicidal agents against non-*albicans Candida* strains, with few (MIC = 1250 μg/mL for *C. albicans* strains ATCC 64129 and ATCC 90028) or no adverse effects on *C. albicans* cells. An exception was noted with *C. albicans* ATCC 90029, which showed susceptibility to COS comparable to that of the NAC strains. These results agree with those of previous works, which have indicated that, in general, most *C. albicans* strains, such as ATCC 90028, have lower sensitivity to chitooligosaccharides and chitosan with varying MWs than non *albicans* species do.^32^

**Table 1:** Minimal Inhibitory Concentration (MIC) and Minimal Lethal Concentration (MLC) of COS Against *Candida* Strains

**strains**	**MIC** (μg/mL)	**MLC** (μg/mL)
*C. albicans* ATCC 10231	N.I.^a^	N.L.^b^
*C. albicans* ATCC 44858	N.I.^a^	N.L.^b^
*C. albicans* ATCC 64129	1250	N.L.^b^
*C. albicans* ATCC 90028	1250	N.L.^b^
*C. albicans* ATCC 90029	156	312
*C. krusei* ATCC 6258	312	312
*C. parapsilosis* ATCC 22019	312	312
*C. parapsilosis* ATCC 90018	312	625
*C. tropicalis* ATCC 750	78	156
*C. tropicalis* ATCC 13803	156	156

a No inhibition of cell growth was observed at the highest concentration tested (1.25 mg/mL).

b No lethal effect was observed at the highest concentration tested (1.25 mg/mL).

The antifungal activity of COS against non-*albicans* strains was further demonstrated through an agar diffusion test using droplets of 0.2% agarose gel containing 0.5% (m/v) COS. In this antifungal assay, the antifungal effects of COS were also compared with those of a commercial ketoconazole-based antifungal cream. As shown in Figure S11, growth inhibition zones were observed around the COS-containing agarose spots (dose = 15 mg) in all non-*albicans* cultures tested, with diameters ranging from 10 mm (*C. krusei* ATCC 6258) to 24 mm (*C. tropicalis* ATCC 13803). Moreover, for the three strains, the mean sizes of the growth inhibition zones caused by COS were comparable (*C. krusei* ATCC 6258 and *C. tropicalis* ATCC 750) or even larger (*C. tropicalis* ATCC 13803) than those produced by the commercial antifungal cream (dose = 12 mg) containing 2% ketoconazole (Figure S11 and Table S1). Ketocanozole is an imidazole derivative that is extensively used in topical formulations, such as shampoos, creams and gels, for the treatment of fungal dermal infections and seborrheic dermatitis.^59,60^ Considering that COSs are as effective as ketoconazole in inhibiting the growth of non-*albicans* strains *in vitro*, these results demonstrate that chitosan oligomers are promising molecules that could be further investigated as potential candidates for the treatment of infections caused by these *Candida* species. Because most studies related to COS activity against *Candida* have focused on *C*. *albicans* strains, we further analyzed the antifungal effects of COS on *C. krusei* (ATCC 6258) and *C. parapsilosis* (ATCC 22019), which are two clinically important non-*albicans* species.^67^

### 3.4 Time–Kill Kinetics Assay

The antifungal effectiveness of COS against *C. krusei* ATCC 6258 and *C. parapsilosis* ATCC 22019 was evaluated via time–kill kinetics assays (Figure 2). When *C. krusei* ATCC 6258 cells were incubated with COS at 312 μg/mL (MIC) or 624 μg/mL (2 × MIC), it took 4 h to kill all the cells, and no viable cells remained after 24 h of exposure at either concentration. COS also significantly reduced (*p* < 0.05) the number of viable *C. krusei* cells when tested at a sublethal concentration (1/2 × MIC), and this effect lasted until 24 h posttreatment. *C. parapsilosis* ATCC 22019 cells were killed more rapidly (within 2 h) at COS concentrations of 312 μg/mL (MIC) and 624 μg/mL (2 × MIC), and fungicidal activity was observed until 24 h after both treatments. At 156 μg/mL (1/2 × MIC), COS promoted a 1-log reduction in CFU/mL of *C. parapsilosis* culture within the first 6 h post-treatment. The growth of the colony resumed from that point on, but the number of viable cells remained significantly lower (*p* < 0.05) until 24 h than that in the untreated culture.

**Figure 2:**
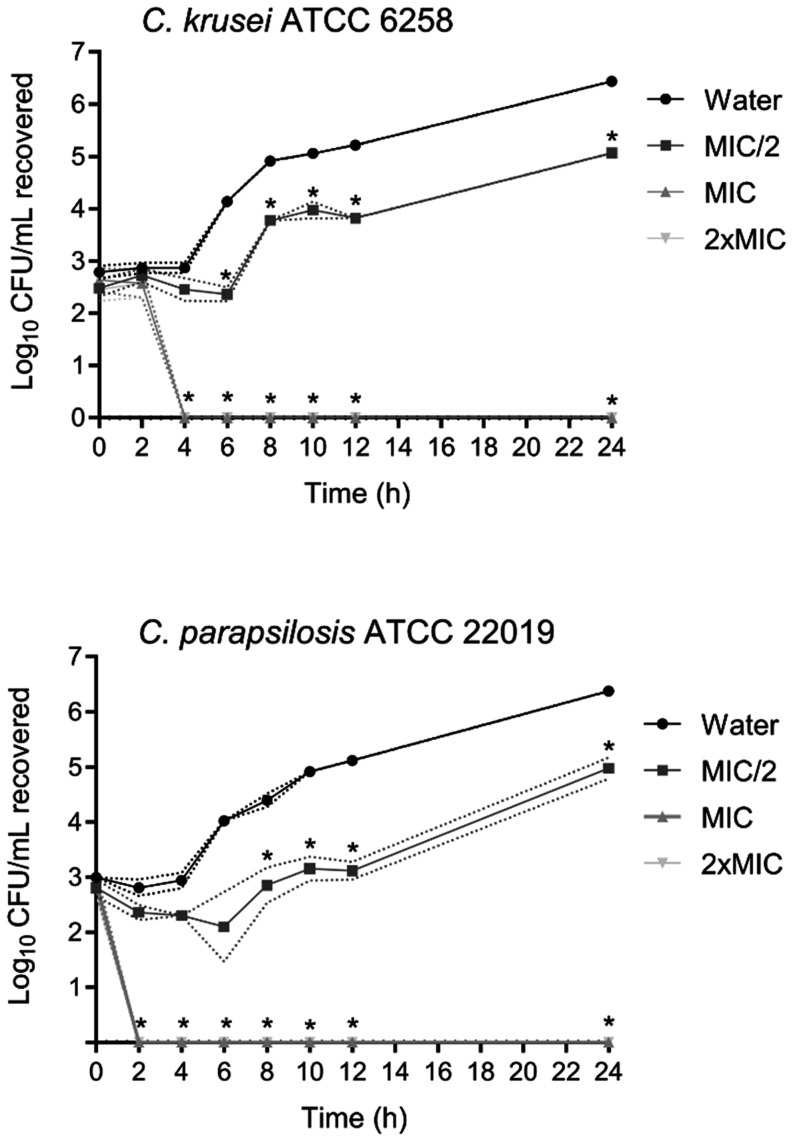
Time–kill kinetics assay of COS activity against *C. krusei* ATCC 6258 and *C. parapsilosis* ATCC 22019. The assays were performed in triplicate (the data are the means ± SDs), as described in the methods section (subsection 2.8.3). For each treatment and control, the means and upper and lower bounds of one SD from the mean are connected by solid and dashed lines, respectively. At each time point, the means that are significantly different (*p* < 0.05; Dunnett’s multiple comparisons test) from those of the control (water) are indicated by asterisks.

### 3.5 Antibiofilm Effects of COS

COS did not inhibit the formation of biofilms by *C. krusei* ATCC 6258 or *C. parapsilosis* ATCC 22019 when tested at three different concentrations (1/2 × MIC, MIC and 2 × MIC) (Figure S12). However, COS at 2 × MIC caused significant (*p* < 0.05) partial disorganization of a preformed biofilm of *C. krusei* ATCC 6258, as evidenced by a reduction of approximately 23.1% in total cell biomass. This effect was similar to that observed in preestablished biofilms of *C. krusei* (ATCC 6258) treated with fluconazole, which promoted a total biomass reduction of 23.7% (Figure S12). The biofilm formed by *C. parapsilosis* ATCC 22019 was more susceptible to COS, which promoted significant (*p* < 0.05) reductions in the total cell biomass of biofilms of approximately 20% (MIC) and 39% (2 × MIC). Conversely, fluconazole had no effect on the preformed biofilm of this strain (Figure S12).

### 3.6 Effects of COS on *Candida* Cell Membrane Integrity, the Production of ROS and Cell Morphology

The fluorescence of *Candida* cells (*C. krusei* ATCC 6258 and *C. parapsilosis* ATCC 22019) exposed to COS (MIC) for 1 h and labeled with PI was much greater than that of untreated cells (Figure 3A), indicating damage to the cell membrane and cell death.^61^ The PI florescence intensity of the COS-treated cells was comparable to or even greater than that of cells treated with 50% ethanol, an antimicrobial agent that increases the fluidity of the plasma membrane and disrupts its physical structure.^62^ Consistent with the fluorescence microscopy images, flow cytometry analysis (Figure S13) revealed that the percentage of dead *C. parapsilosis* (ATCC 22019) cells after COS treatment (PI+, SSC+: 79.74%) was equivalent to the proportion of dead cells after incubation with 50% ethanol (PI+, SSC+: 80.05%). For *C. krusei* (ATCC 6258), the subpopulation of dead cells (PI+, SSC+) represented 89.26%, whereas the subpopulation of nonviable cells (PI+, SSC+) after 50% ethanol treatment was 61.32%.

**Figure 3:**
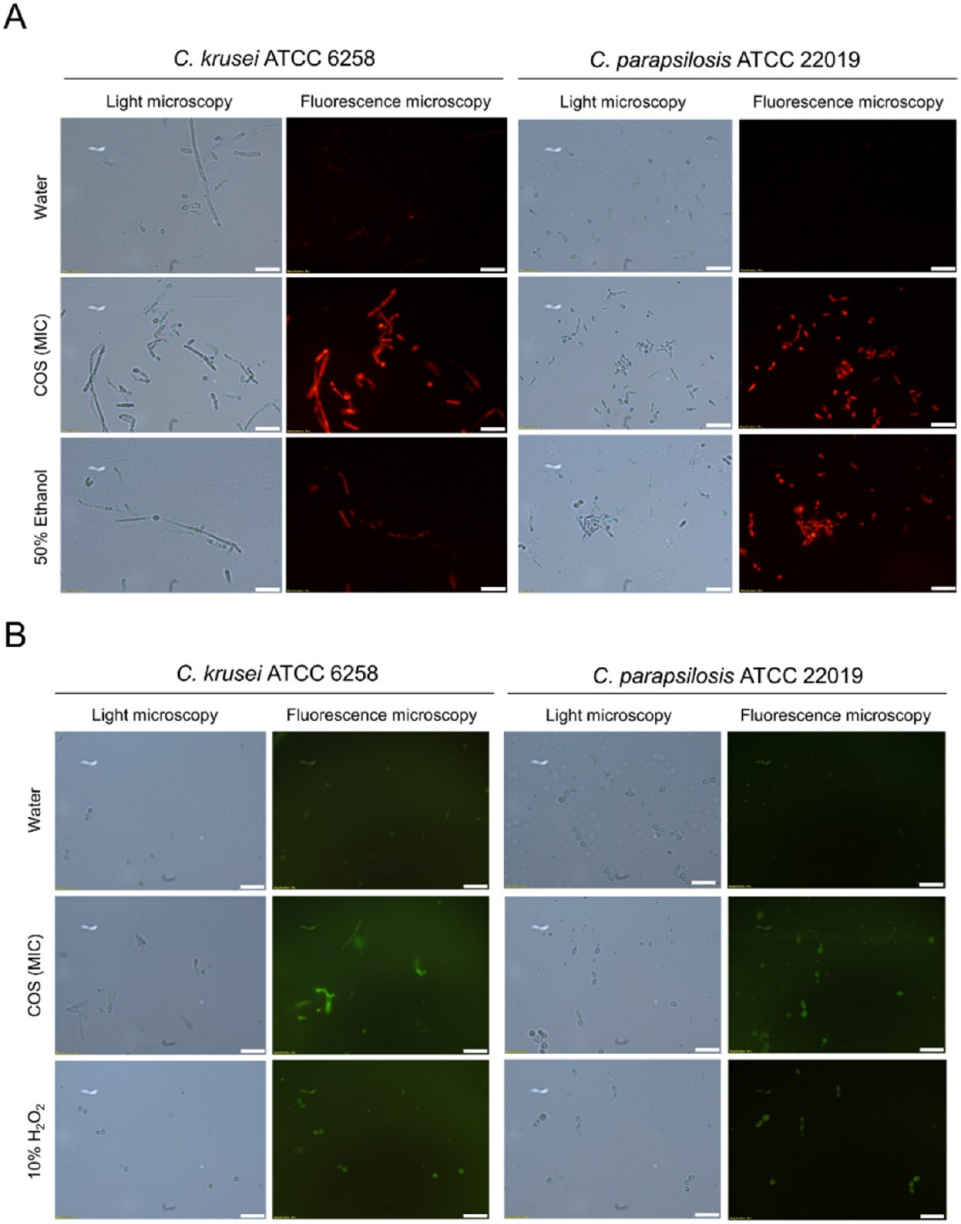
Cell permeability and nuclear DNA staining by PI (A) and detection of ROS generation by DCFH2-DA (B) in *Candida* cells treated with COS. *C. krusei* ATCC 6258 and *C. parapsilosis* ATCC 22019 cells were treated with COS (MIC) and subjected to PI and DCFH2-DA staining, as described in the methods section (subsections 2.9 and 2.10). The cells treated with water, 50% (v/v) ethanol or 10% (v/v) H_2_O_2_ were included as controls. The scale bar represents 20 μm.

In addition to promoting damage to the cell membrane, which compromises cell viability, COS (MIC) also causes changes in the cellular redox state of *Candida* strains (*C. krusei* ATCC 6258 and *C. parapsilosis* ATCC 22019), increasing total ROS production after 1 h of treatment, as evidenced by the use of the cell-permeable fluorogenic redox probe DCFH2-DA (Figure 3B). The fluorescence of ROS-positive cells treated with COS was brighter than that of cells treated with H_2_O_2_, an oxidant that, at supraphysiological concentrations, increases the levels of ROS, including the mitochondrial superoxide anion (O_2_^•–^), causing growth arrest and cell death.^63,64^

The cell morphology of *Candida* strains (*C. krusei* ATCC 6258 and *C. parapsilosis* ATCC 22019) treated with COS (MIC) was analyzed via SEM (Figure 4). Compared with untreated cells, which had an even, smooth surface as well as a normal cell size and subspherical or oval shape, most cells treated with COS were rounder and presented a rough, irregular surface and overall loss of cell integrity. The overall appearance of the heavily deformed, collapsed *Candida* cells treated with COS resembled that observed in cell samples treated with fluconazole (included for comparison, Figure 4), an azole that inhibits lanosterol 14α-demethylase (CYP51), which compromises the biosynthesis of ergosterol and fungal membrane integrity, leading to blockage of cell reproduction or cell death.^65^ The morphological alterations caused by COS in *Candida* cells can be a consequence of altered cell wall architecture and loss of cell membrane integrity, which might be explained by direct electrostatic interactions of the positively charged GlcN units of the chitosan oligomers with negatively charged components of the fungal cell surface. The disruption of membrane integrity could promote increased cellular permeability, leading to leakage of intracellular constituents and death of *Candida* cells treated with COS. Moreover, the increased ROS levels in yeast cells exposed to COS can directly oxidize biological macromolecules, such as proteins, nucleic acids and lipids, thus contributing to cell membrane damage, increased permeability and ultimately cell death.^66^ Indeed, different classes of antifungal agents, such as the azoles miconazole and itraconazole, the polyene amphotericin B, animal and plant antimicrobial peptides and bacterial lipopeptides, act by increasing ROS production and triggering apoptosis in *Candida* species.^67–70^

**Figure 4:**
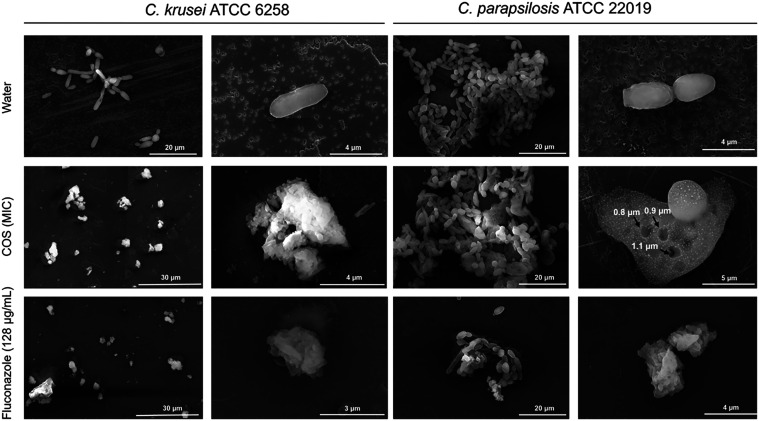
SEM micrographs of *Candida* cells treated with COS. *C. krusei* ATCC 6258 and *C. parapsilosis* ATCC 22019 cells were treated with COS (MIC) and observed via SEM, as described in the methods section (subsection 2.13). Cells treated with water and fluconazole (128 μg/mL) were included as controls. Round areas on the cell surface of *C. parapisilosis* treated with COS were observed and are indicated by arrows (the number next to each arrow represents the diameter of each area). This morphological feature probably reflects the damage caused by COS to target cells. The scale bars represent the sizes shown in parentheses as follows: water, left images (30 μm); water, right images (4 μm); COS (30, 4, 20, and 5 μm, from left to right, respectively); fluconazole (30, 3, 20, and 4 μm, from left to right, respectively).

### 3.7 Effects of Ionic Strength and pH on the Anti-*Candida* Activity of COS

To investigate whether the electrostatic interactions of positively charged COS with the negatively charged components of the yeast cell membrane might be important for its antifungal activity, MIC values were determined by growing the cells at acidic pH (5.5) in the presence of varying concentrations of NaCl (Figure 5A,B). In another experiment, the MIC was determined at different pH values (4.5, 5.5 and 7.5) in the absence of NaCl (Figure 5C). To assess the tolerance/sensitivity of the target fungal cells to NaCl, their growth in culture media with different salt concentrations was first determined. The growth of *C. krusei* ATCC 6258 and *C. parapsilosis* ATCC 22019 was not significantly affected (*p* > 0.05) in the presence of 0.15 or 0.3 M NaCl. Higher concentrations of NaCl (0.45 and 0.6 M) caused significant (*p* < 0.05) growth reduction in both cell lines, with *C. parapsilosis* being more tolerant than *C. krusei* to the highest (0.6 M) salt concentration tested. This greater salt tolerance of *C. parapsilosis* agrees with previous data reported in the literature.^71^

**Figure 5:**
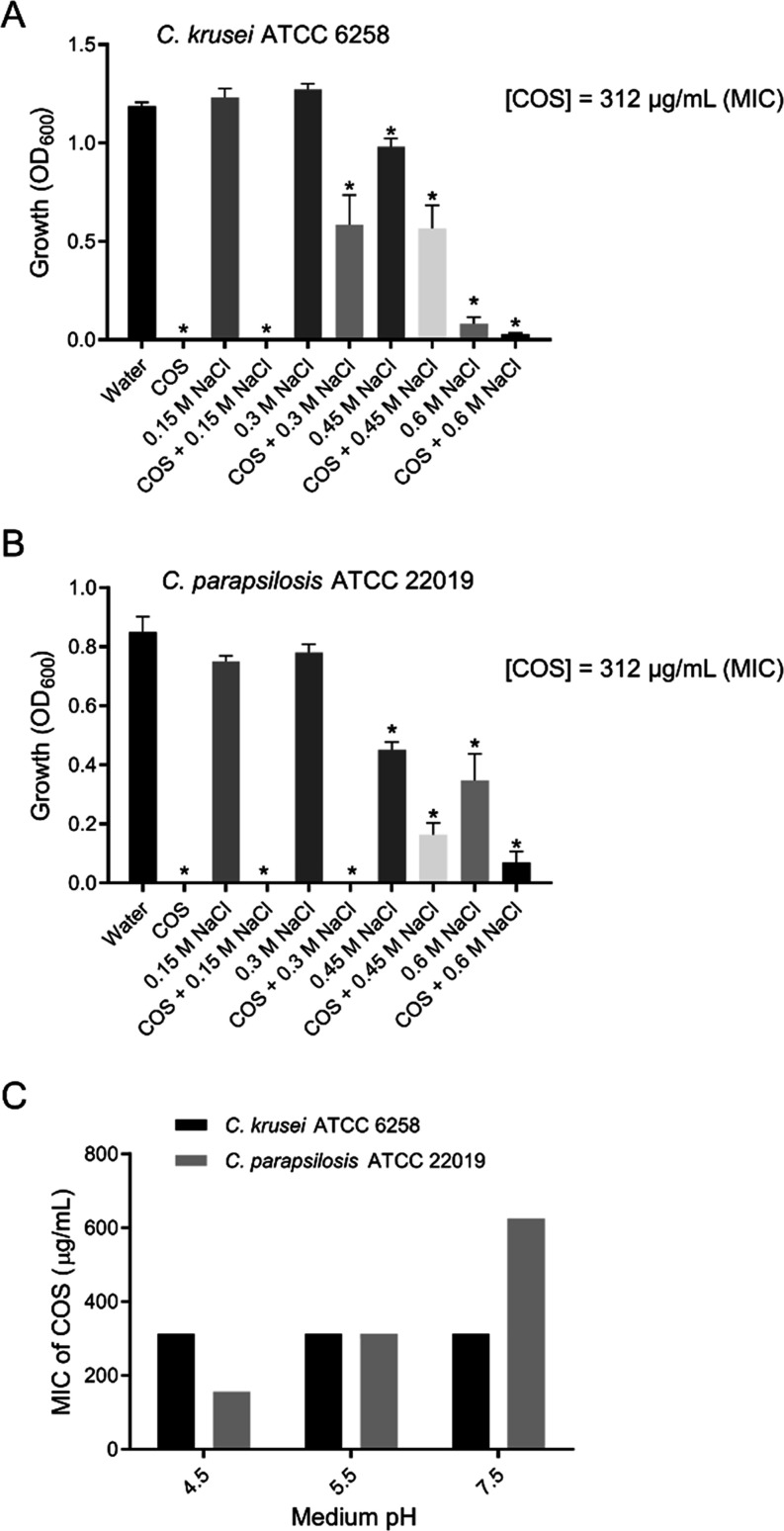
Effects of ionic strength (A and B) and pH (C) on the antifungal activity of COS against *C. krusei* and *C. parapsilosis*. The effects of different concentrations of NaCl (A and B) and different pH values of the culture medium (C) on the antifungal activity of 312 μg/mL COS (MIC) against *C. krusei* ATCC 6258 and *C. parapsilosis* ATCC 22019 were determined through a broth microdilution assay, as described in the methods section (subsection 2.8.1). The assays were performed in triplicate (data are means ± SDs) and means that were significantly different (*p* < 0.05; Dunnett’s multiple comparisons test) are indicated by asterisks.

When *Candida* strains (*C. krusei* ATCC 6258 and *C. parapsilosis* ATCC 22019) were cultivated in the presence of 312 μg/mL COS (MIC) and NaCl at different concentrations, the antifungal activity of the chitosan oligomers significantly (*p* < 0.05) decreased at relatively high salt concentrations. The minimum NaCl concentration able to partially reverse the antifungal action of COS was 0.3 M (for *C. krusei* ATCC 6258) and 0.45 M (for *C. parapsilosis* ATCC 22019). At these salt concentrations, the inhibition of yeast growth caused by COS was approximately 45.9% (for *C. krusei* ATCC 6258) and 36.4% (for *C. parapsilosis* ATCC 22019) (Figure 5A,B). Furthermore, variation in the medium pH (4.5, 5.5 and 7.5) alone did not affect the MIC of COS against *C. krusei* ATCC 6258 cells (312 μg/mL) (Figure 5C). Even at physiological pH (7.4–7.5), low-molecular-weight COS might still have a positive charge.^56,57^ For example, the zeta potential of a COS with 10 GlcN units varied from 45 mV at pH 4.0 to 10 mV at pH 8.0. Therefore, for *C. krusei* ATCC 6258, the number of COS – NH_3_^+^ groups is not a critical feature for antifungal activity, as the MIC value was the same regardless of the pH (4.5, 5.5 or 7.5) at which the assay was performed. Conversely, the MIC of COS against *C. parapsilosis* ATCC 22019 was pH dependent, with antifungal activity increasing as the pH decreased, indicating that the presence of more −NH_3_^+^ groups favors binding to yeast cells, thereby causing greater growth inhibition (Figure 5C). Thus, the MICs of COS for *C. parapsilosis* ATCC 22019, as assessed at pH 4.5 (156 μg/mL), were approximately 4-fold and 2-fold lower than those determined at pH 7.5 (625 μg/mL) and 5.5 (312 μg/mL), respectively. The differences in COS susceptibility between these *Candida* strains are likely associated, at least in part, with changes in membrane lipid composition and membrane fluidity, in parallel with what has been observed for yeast strains susceptible to antifungal drugs that target fungal membrane homeostasis, such as azoles.^72^ The influence of ionic strength and pH on the anti-*Candida* activity of COS indicates that electrostatic interactions between the positively charged chitooligosaccharides and the negatively charged surface of the target cells play a role in their antifungal activity, but nonionic interactions are also involved. Similarly, sequestration of glycodeoxycholic acid and other bile salts by low-molecular-weight chitosan oligomers (with average degrees of polymerization and deacetylation of 10 and 90%, respectively) involves both ionic and nonionic interactions.^57^

### 3.8 Assessment of the Antifungal Activity of COS toward Filamentous Fungi

The effects of COS (concentrations ranging from 7.8 to 1000 μg/mL) on the spore germination and mycelial growth of 8 filamentous fungi belonging to 4 different genera (*Colletotrichum*, *Fusarium*, *Mucor* and *Penicillium*) were also evaluated. Significant (*p* < 0.05) inhibition of *in vitro* mycelial growth was observed toward *F. lateritium* (Figure S14D), *F. oxysporum* (Figure S14E) and *F. solani* (Figure S14F), and the antifungal effect was dose dependent. The half-maximal inhibitory concentrations (IC_50_) were 476.9 μg/mL (for *F. lateritium*), 298 μg/mL (for *F. oxysporum*) and 316.5 μg/mL (for *F. solani*) after 48 h of COS treatment. Complete inhibition of *F. oxysporum* and *F. solani* mycelial growth was attained at the highest COS concentration (1 mg/mL) tested. In contrast, the three species of the genus *Colletotrichum* (*C. gloeosporioides*, *C. lindemuthianum* and *C. scovillei*) were the most resistant to COS treatment, with no growth inhibition observed at the highest COS concentration (1000 μg/mL) tested (Figures S14A-S14C). Two other species, *M. circinelloides* (Figure S14G) and *P. decumbens* (Figure S14H), were susceptible to COS. The minimum COS concentrations that significantly (*p* < 0.05) reduced mycelial growth were 250 μg/mL (for *P. decumbens*) and 1000 μg/mL (for *M. circinelloides*). However, no complete inhibition of these two fungi was attained by any of the COS concentrations. Conversely, COS at lower concentrations (31.25 and 62.5 μg/mL) significantly (*p* > 0.05) promoted an increase in the mycelial biomass of *M*. *circinelloides*, a growth stimulating effect of chitosan oligomers that was also observed for other filamentous fungi, which apparently use low-molecular-weight COS as nutrients.^73^ Therefore, the antifungal effectiveness of COSs depends on their concentration and the target species tested.

*Fusarium oxysporum* and *F. solani*, together with *F. psedogramanium* and *F. graminearium*, are considered the most important plant pathogenic *Fusarium* species worldwide, causing many diseases, such as blights, wilts, cankers and rots, in more than 150 crop species.^74,75^ The antifungal mechanism of COS was further examined in these two important phytopathogens. At the IC_50_, COS promoted permeabilization of the cell membrane and induced an increase in total intracellular ROS production in both *F. oxysporum* and *F. solani*, as indicated by fluorescence microscopy of SYTOX Green- and DCFH2-DA-stained fungal cells (Figure 6A,B).

**Figure 6:**
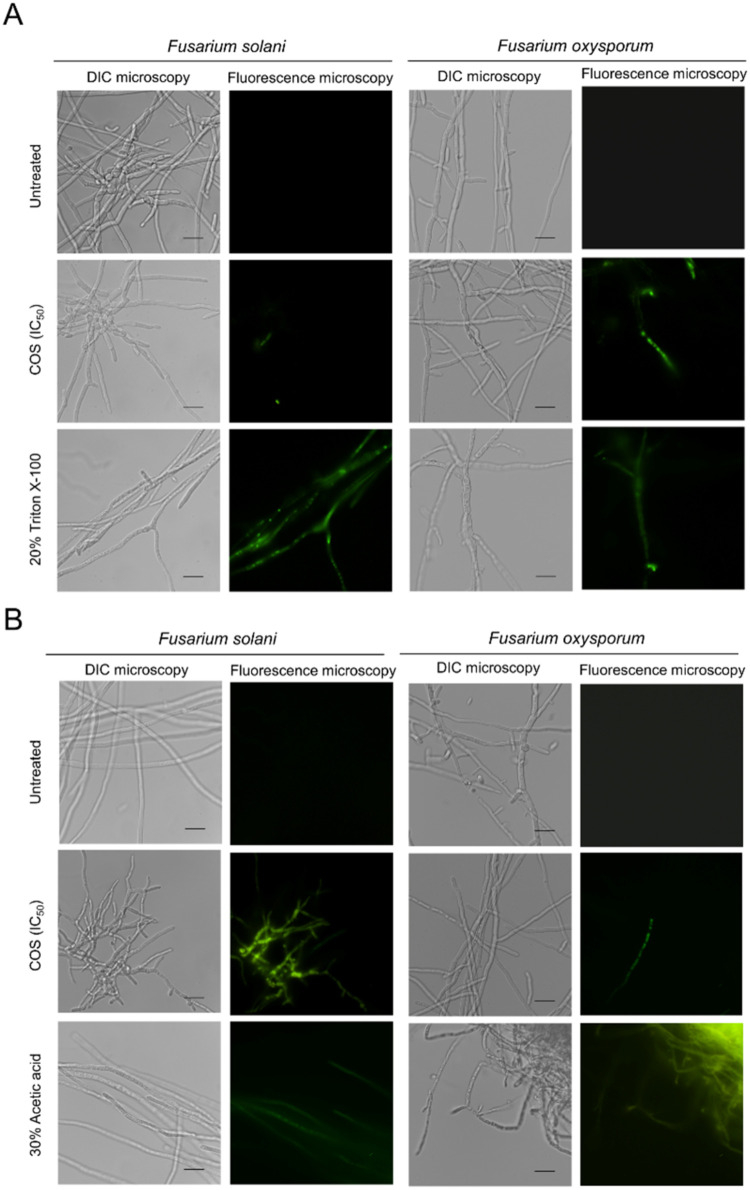
Cell permeability and nuclear DNA staining by SYTOX Green (A) and detection of ROS generation by DCFH2-DA (B) in *Fusarium* cells treated with COS. Mycelia of *F. oxysporum* and *F. solani* were treated with COS (IC_50_) and subjected to SYTOX Green and DCFH2-DA staining, as described in the methods section (subsection 2.12). Untreated mycelia and mycelia treated with 20% (v/v) Triton X-100 (A) and 30% (v/v) acetic acid (B) were included as controls. The scale bar represents 20 μm.

SEM analysis revealed that COS also caused morphological alterations, as depicted in Figure 7. The hyphae of *F. oxysporum* treated with COS appeared thinner and shorter, with amorphous material deposited on the cell wall. There was also evident disruption of membrane continuity, indicating loss of cell integrity. Conversely, the mycelia of *F. solani* treated with COS presented an abnormal branching pattern, with excessive and irregular hyphal production, which was covered by abundant extracellular material. These morphological alterations were not observed in the untreated mycelia of either species. The antifungal effectiveness of the low-molecular-weight COSs (DPs of 2–4) against *Fusarium* reported in this work agrees with previous works that described the inhibition of *F. oxysporum* strains by enzymatically generated, well-defined GlcN oligomers with DPs of 2–4 ^24^ and 3–5.^26^ Therefore, COS might be alternative fungicide molecules for the control of plant diseases caused by pathogenic *Fusarium* species, which are resistant to major classes of commercial fungicides, including triazoles, phenylpyrroles and benzimidazoles, in various parts of the world.^76^

**Figure 7:**
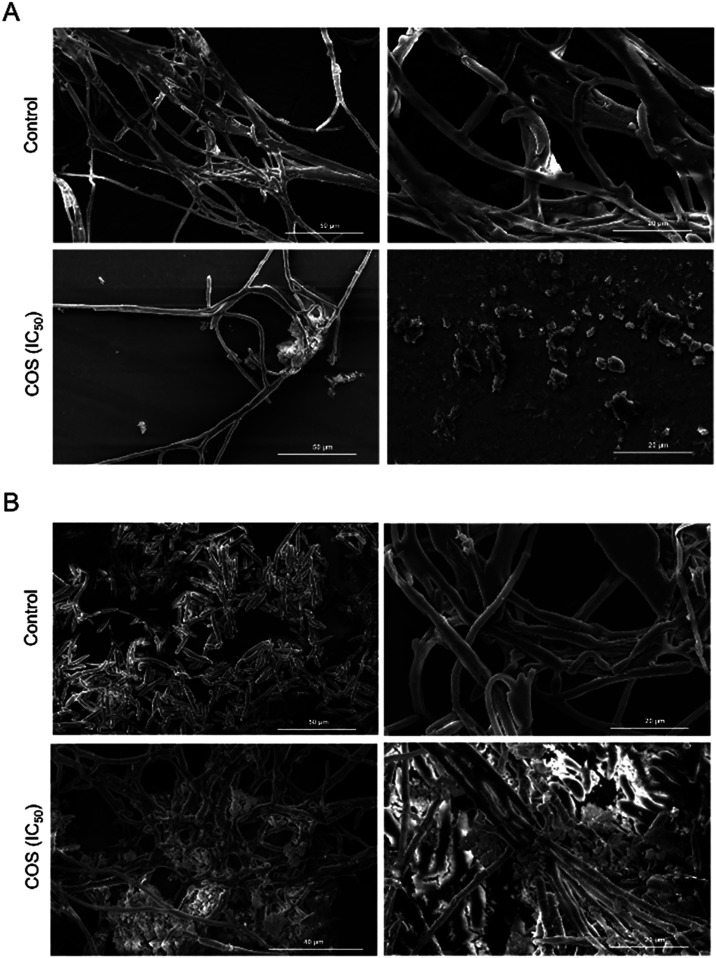
SEM micrographs of *Fusarium oxysporum* (A) and *F. solani* (B) treated with COS. Mycelia of *F. oxysporum* (A) and *F. solani* (B) were treated with COS (IC_50_) and observed via SEM, as described in the methods section (subsection 2.13). Untreated mycelia were included as controls. Scale bars represent 50 μm (panel A, control, left image; panel A, COS, left image; panel B, control, left image), 40 μm (panel B, COS, left image) and 20 μm (panels A and B, control, right images; panels A and B, COS, right images).

## 4 Conclusions

In this work, a mixture of low-molecular-weight (*M*_w_ < 700 Da) chitosan oligomers (GlcN_2–4_) and the monosaccharide GlcN were generated via the enzymatic hydrolysis of polymeric chitosan with the bacterial chitosanase *Cv*Csn46. The antifungal activity of the COS preparations toward 4 species of *Candida* was determined and shown to be genotype specific, with non-*albicans* strains of 3 species (*C. krusei*, *C. parapsilosis* and *C. tropicalis*) being more susceptible than *C. albicans*. Among the *C. albicans* strains tested, only one (*C. albicans* ATCC 90029) was susceptible to COS. These findings indicate a differential antifungal effect, with COS being more effective against NAC species and selectively active against certain *C. albicans* strains. Among the filamentous fungi tested, COS was more effective at inhibiting the growth of two plant pathogens, *Fusarium oxysporum* and *F. solani*, whereas the growth of other species was unaffected or even stimulated by COS at certain concentrations. The results obtained here show that the antifungal mode of action of COS involves disruption of the cell membrane structure, membrane permeabilization and ROS production, leading to morphological alterations and cell death. Experimental evidence showed that although ionic interactions contribute to COS antifungal activity, nonionic interactions are a relevant structural feature involved in the binding of COS to target microorganisms, which ultimately leads to cell death.

## Supplementary Material



## Notes

ESI-MS and ESI-MS/MS spectra; Ramachandran plots, model quality estimation, structural superpositions and structural alignment; FTIR spectra; antifungal activity assay (agar diffusion test) and antibiofilm assay against *Candida* strains; flow cytometric analysis of *Candida* cells stained with propidium iodide; antifungal activity assays against filamentous fungi (PDF)

## #

Departamento de Genética, Faculdade de Medicina de Ribeirão Preto, Universidade de São Paulo, Ribeirão Preto, SP 14049–900, Brazil

## $

Departamento de Bioquímica, Instituto de Química, Universidade de São Paulo, São Paulo, SP 05508–000, Brazil
