# Supplementary material for: Targeting Non-albicans Candida Strains and Phytopathogenic Fusarium Species with Chitooligosaccharides: Insights into the Antifungal Mechanism
Source: ACS Omega. 2025 Dec 6;10(50):61852–66. doi: 10.1021/acsomega.5c08432 (PMC12750198; doi:10.1021/acsomega.5c08432)
Supplement: Supplementary file 1 [file ao5c08432_si_001.pdf]

# Supporting Information

# Targeting Non-*albicans Candida* Strains and Phytopathogenic *Fusarium* Species with Chitooligosaccharides: Insights into the Antifungal Mechanism

Mayara I. G. Azevedo [a#], Nadine M. S. Araujo [a], Filipe A. Vieira [a], Milena B. Cherene [b], Bruno R. S. Queiroz [a$], Daniele O. B. Sousa [a], Celso S. Nagano [c], Rômulo F. Carneiro [c], Valdirene M. Gomes [b], Rafael G. G. Silva [d], José E. Monteiro-Júnior [d], Cosme S. Sousa [e], Bruno L. Sousa [e], Thalles B. Grangeiro [d*]

[a] Programa de Pós-Graduação em Bioquímica, Departamento de Bioquímica e Biologia Molecular, Centro de Ciências, Universidade Federal do Ceará, Campus do Pici, Fortaleza, CE, 60440-900, Brasil

[b] Laboratório de Fisiologia e Bioquímica de Microrganismos, Universidade Estadual do Norte Fluminense Darcy Ribeiro, Campos dos Goytacazes, RJ, 28013-602, Brasil

[c] Departamento de Engenharia de Pesca, Centro de Ciências Agrárias, Universidade Federal do Ceará, Campus do Pici, Fortaleza, CE, 60440-900, Brasil

[d] Departamento de Biologia, Centro de Ciências, Universidade Federal do Ceará, Campus do Pici, Fortaleza, CE, 60440-900, Brasil

[e] Faculdade de Filosofia Dom Aureliano Matos, Universidade Estadual do Ceará, Limoeiro do Norte, CE, 62930-000, Brasil

*Corresponding author (email: thalles@ufc.br)

Present Addresses:

[#] M.I.G.A.: Departamento de Genética, Faculdade de Medicina de Ribeirão Preto, Universidade de São Paulo, Ribeirão Preto, SP, 14049-900, Brazil

[$] B.R.S.Q.: Departamento de Bioquímica, Instituto de Química, Universidade de São Paulo, São Paulo, SP, 05508-000, Brazil

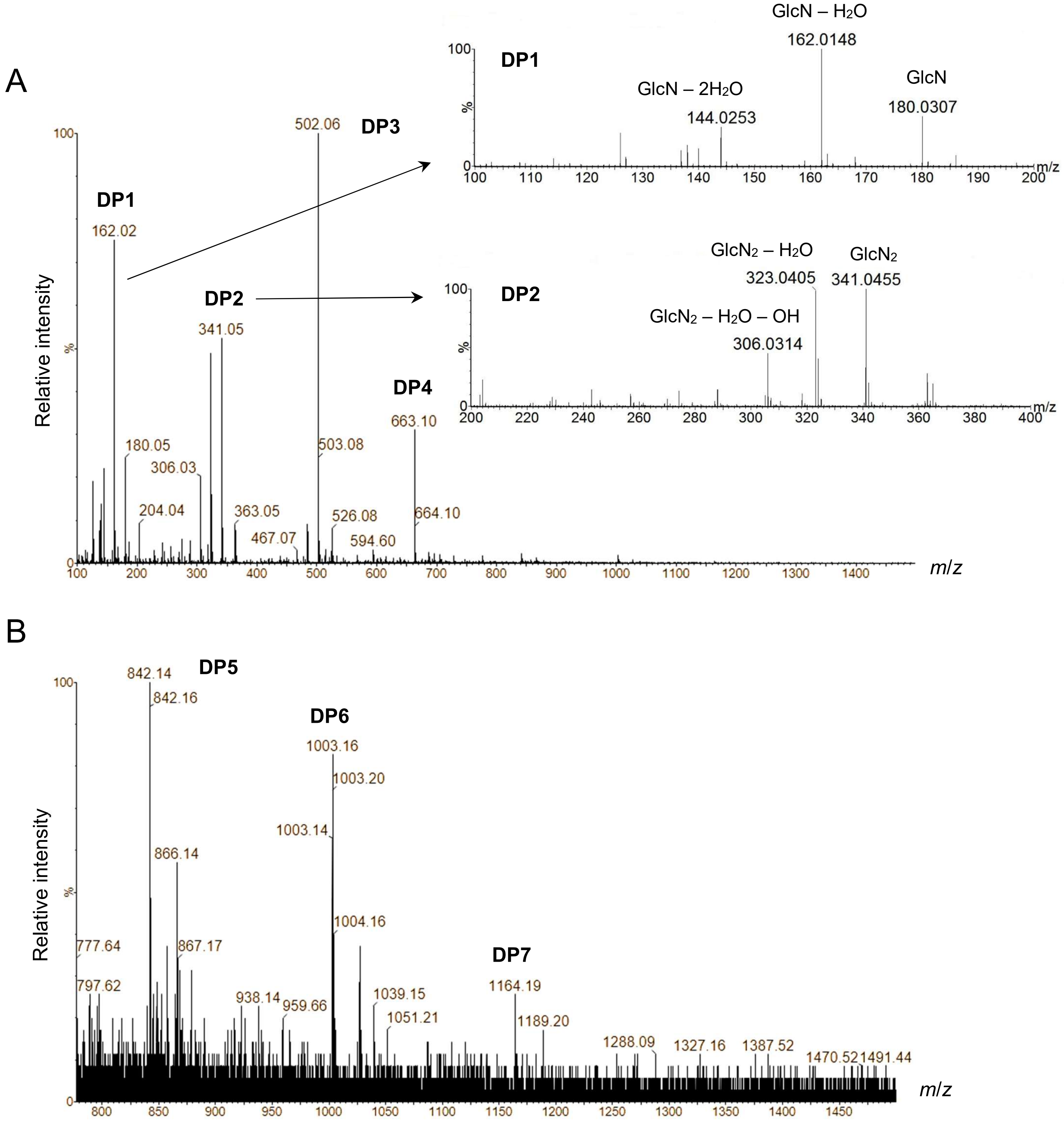


Figure S1. MS analysis of COS produced by enzymatic hydrolysis. ESI–MS primary mass spectrum (positive ion mode; *m*/*z* 100-1450) of the enzymatic products released from colloidal chitosan incubated with *Cv*Csn46 (**A**) and the ESI–MS zoom scan spectrum of the cluster ions at *m*/*z* 750-1500 (**B**).

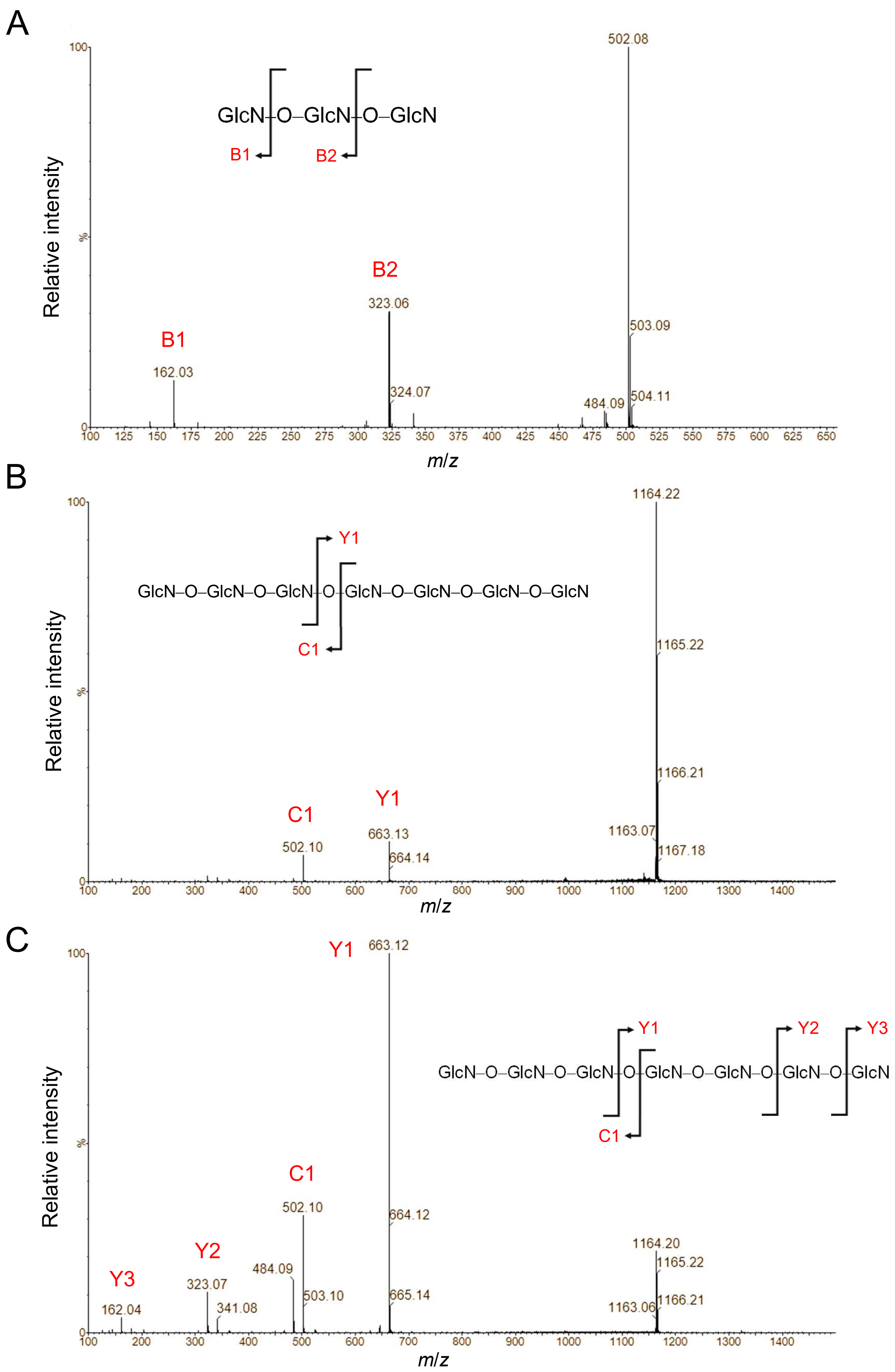


Figure S2. ESI–MS/MS low-energy fragmentation spectra. MS/MS fragmentation spectra of cluster ions at *m/z* 502.08 [$(GlcN)_3$; calculated mass = 501.5 Da] (**A**) and 1164.2 [$(GlcN)_7 + H_2O$; calculated mass = 1164.1 Da] (**B, C**).

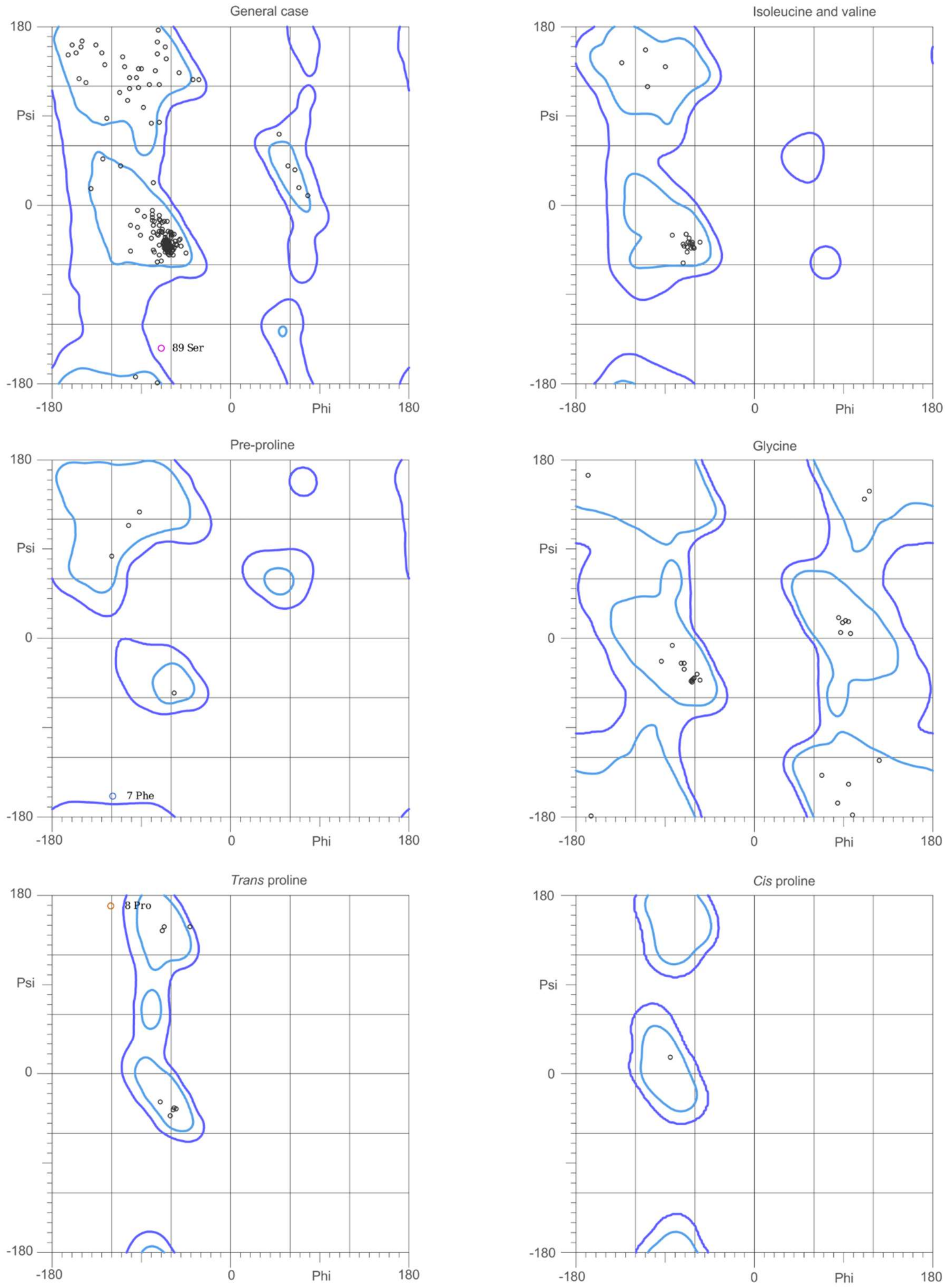


Figure S3. Ramachandran plot showing the main chain dihedral angles (ϕ, ψ) from all residues of the three-dimensional model of the catalytic domain of *Cv*Csn46 in the open conformation. The plot was obtained by submitting the model atomic coordinates to the MolProbity server (http://molprobity.biochem.duke.edu/) [1]. The percentages of all residues in the favored and allowed regions were 96.1% (247/257) and 98.8% (254/257), respectively. Only 3 outliers (1.2%) were observed. The Ramachandran Z score (Rama-Z) value was |Z| = 1.19 ± 0.49.

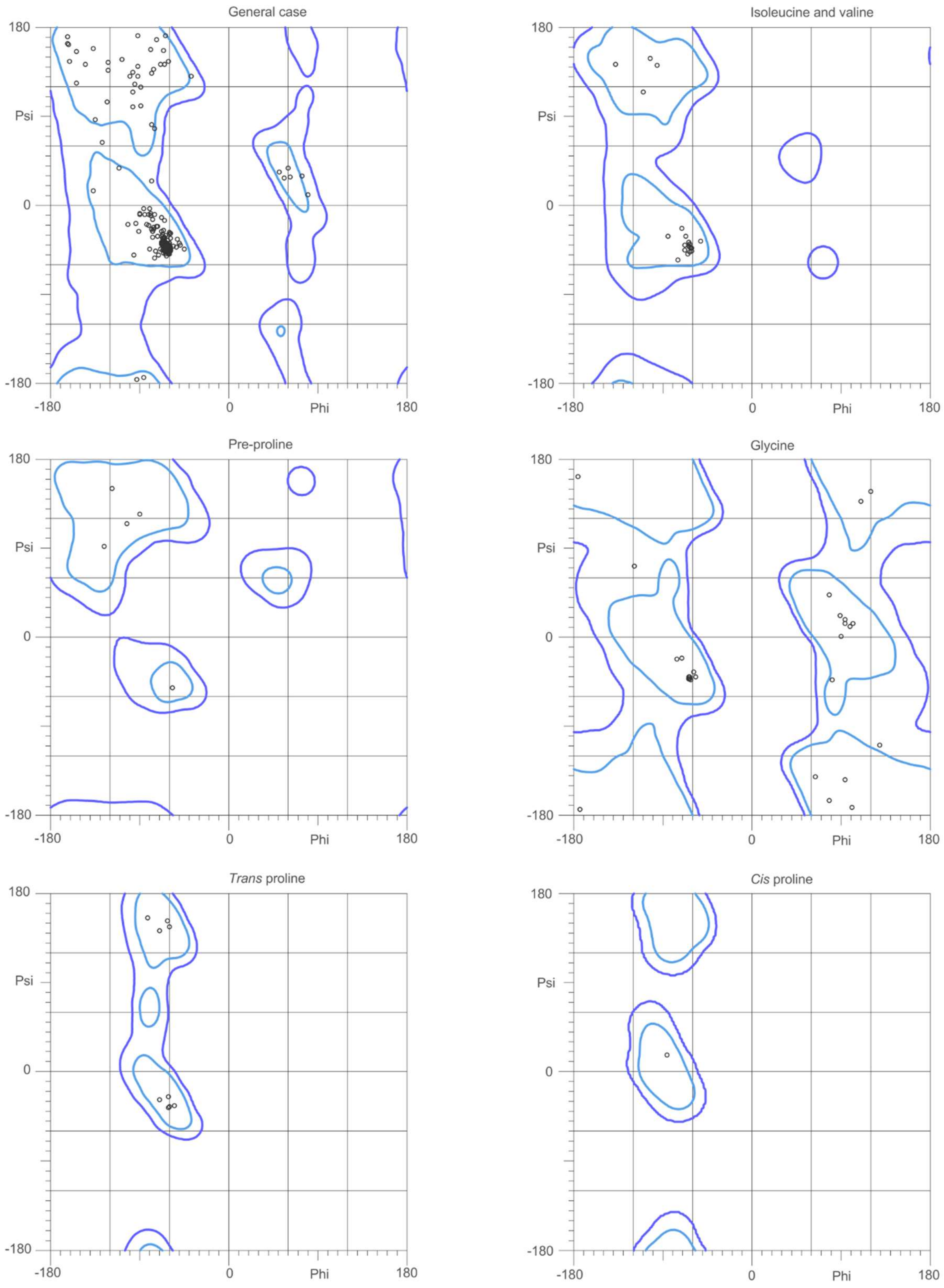


Figure S4. Ramachandran plot showing the main chain dihedral angles (ϕ, ψ) from all residues of the three-dimensional model of the catalytic domain of *Cv*Csn46 in the closed conformation. The plot was obtained by submitting the model atomic coordinates to the MolProbity server (http://molprobity.biochem.duke.edu/) [1]. The percentages of all residues in the favored and allowed regions were 97.3% (250/257) and 100.0% (257/257), respectively. There were no outliers. The Ramachandran Z score (Rama-Z) value was |Z| = 1.15 ± 0.47.

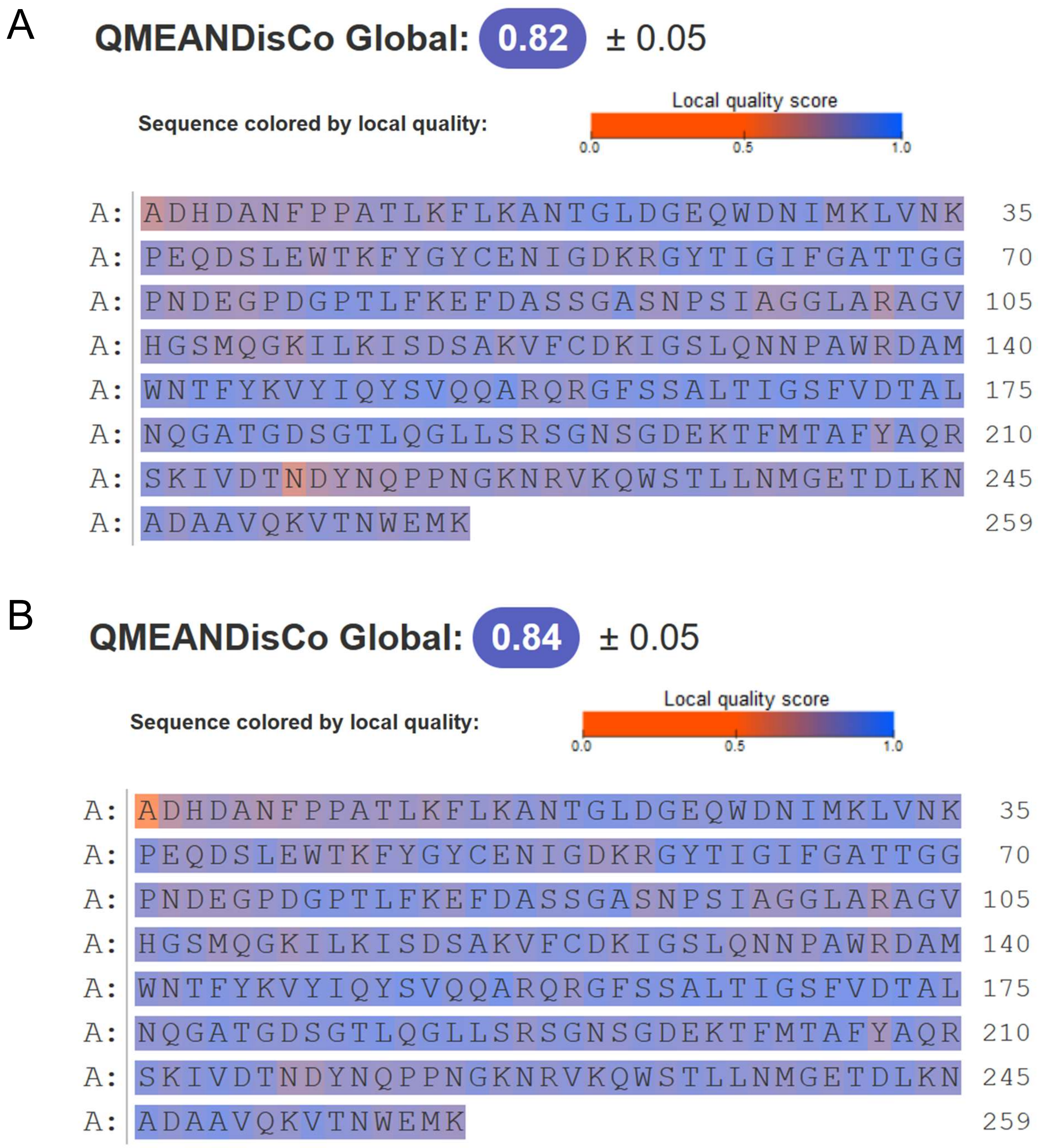


Figure S5. Quality estimation of the three-dimensional models of the GH46 catalytic domain (259 amino acid residues) of *Cv*Csn46. A quality assessment of the homology models of *Cv*Csn46 in the open (**A**) and closed (**B**) conformations was performed using the QMEANDisCo method [2], which is available through the Structure Assessment tool (https://swissmodel.expasy.org/assess, accessed on 30 January 2025) of the SWISS-MODEL server [3]. In each panel, the QMEANDisCo global value and the provided error estimate are shown above the model amino acid sequence, in which the residues are colored by local quality scores as a red-to-blue color gradient.

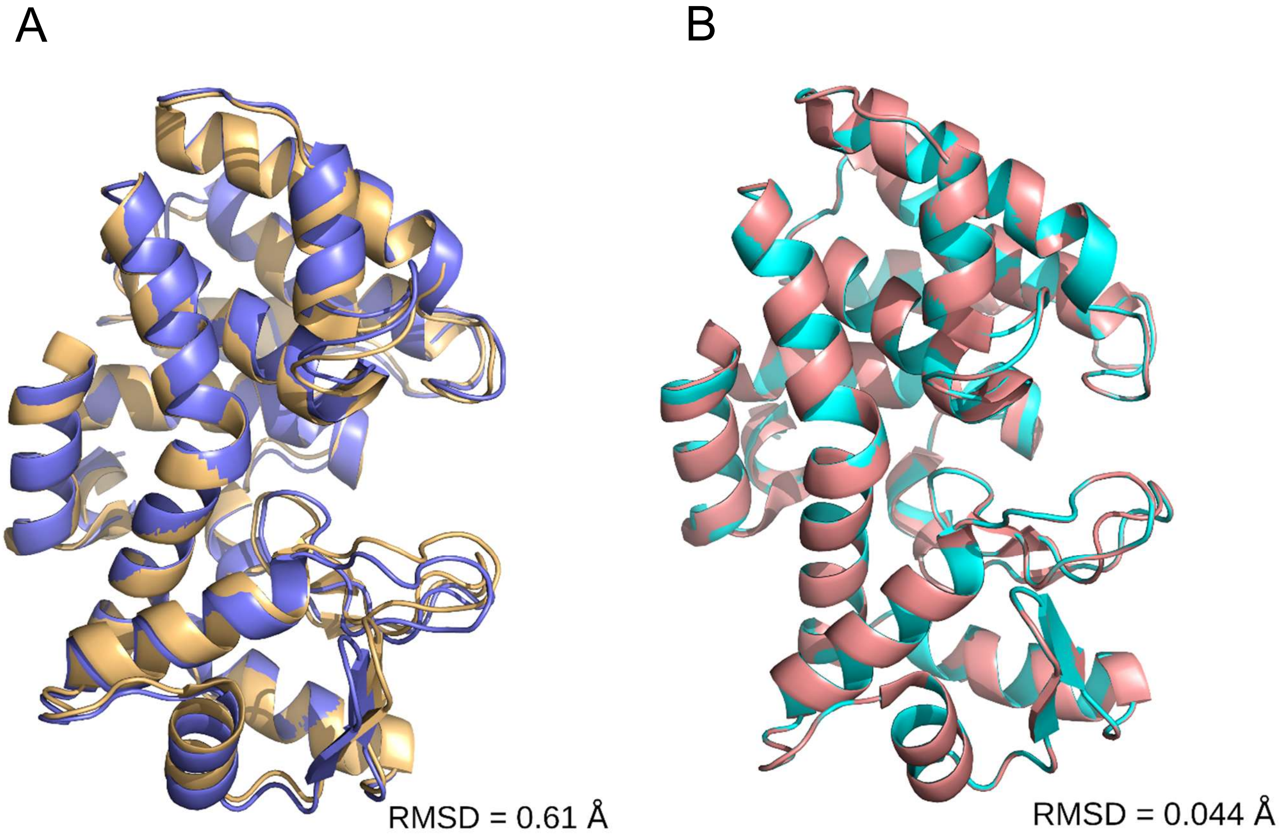


Figure S6. Pairwise structural superpositions of the open and closed structure models of *Cv*Csn46 and X-ray crystallographic structures of GH46 bacterial chitosanases. The open (**A**; slate) and closed (**B**; cyan) three-dimensional models of the CatD of *Cv*Csn46 were superimposed with the X-ray crystallographic structures of the chitosanases Csn-PD from *Paenibacillus dendritiformis* (**A**; apo-form structure, light orange; PDB ID: 7XH0) and CsnMHK1 from *N. circulans* (**B**; substrate-bound form structure, deep salmon; PDB ID: 5HWA). The 3D models and X-ray crystallographic structures are shown as cartoon ribbon diagrams.

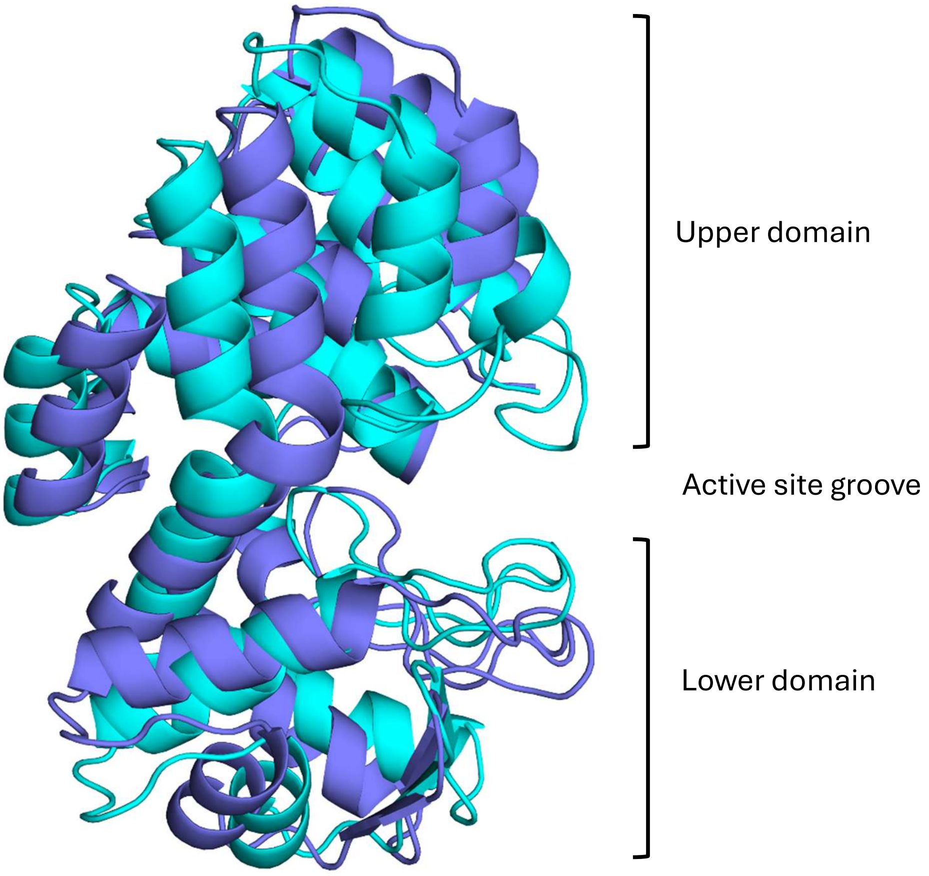


Figure S7. Pairwise structural superposition of the open and closed structural models of *Cv*Csn46. The 3D models of the open (slate) and closed (cyan) forms of *Cv*Csn46 were aligned (RMSD = 2.082 Å) and are shown as cartoon ribbon diagrams. The 3D models were generated by homology modeling using as templates the X-ray crystallographic structures of the apo-form (PDB ID: 1QGI) [4] and substrate-bound form (PDB ID: 5HWA) [5] of the chitosanase CsnMHK1 from *N. circulans*.

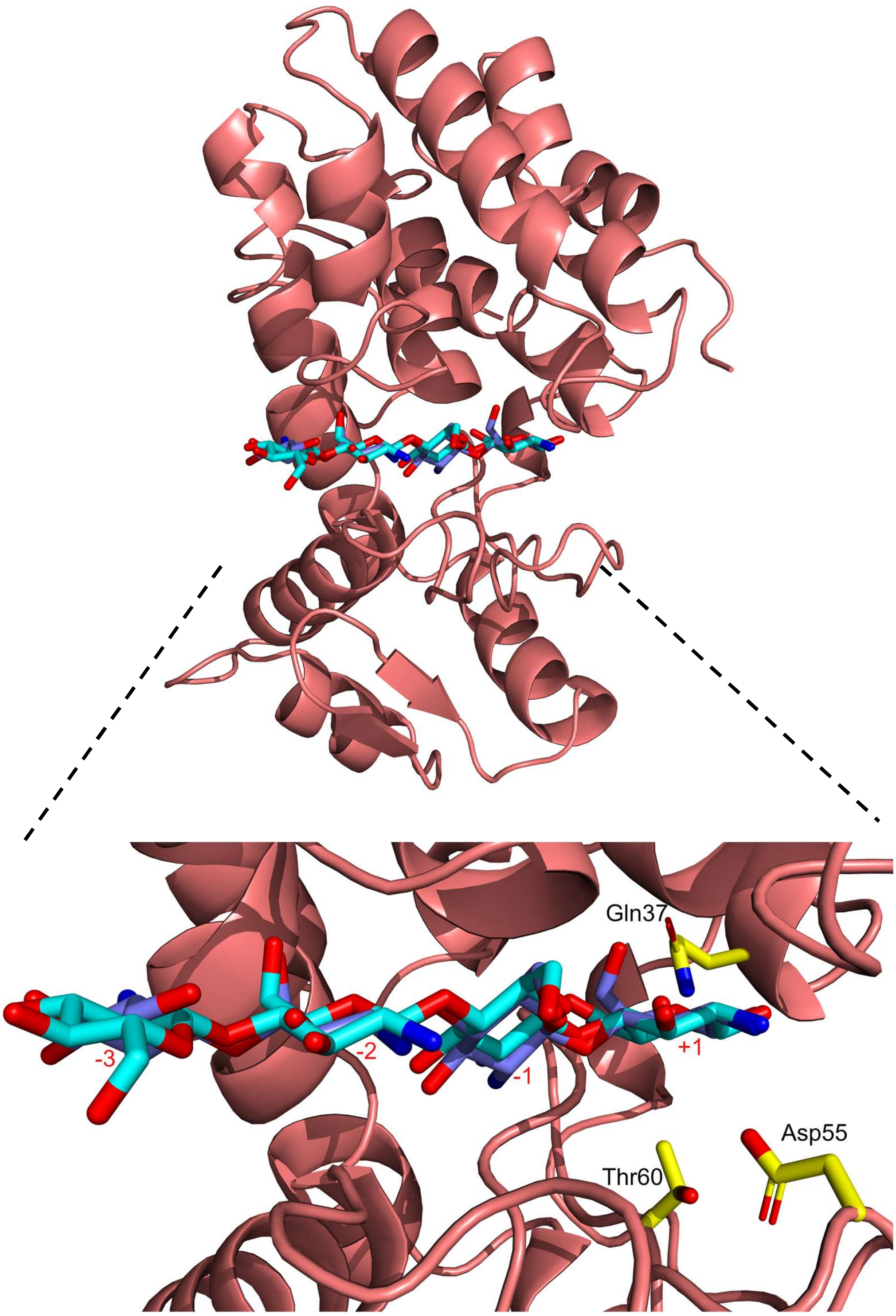


Figure S8. Docking validation by redocking a cocrystal ligand (chitotetraose) in the substrate-binding cleft of CsnMHK1 from *N. circulans*. The ligand crystallized with the E37Q mutant CsnMHK1 (PDB ID: 5HWA) [5] is shown in slate, whereas the ligand that was redocked is shown in cyan. The RMSD between the crystalline and the docked ligands was 1.75 Å. The side chains of the catalytic residues are shown as sticks. N and O atoms are colored blue and red, respectively.

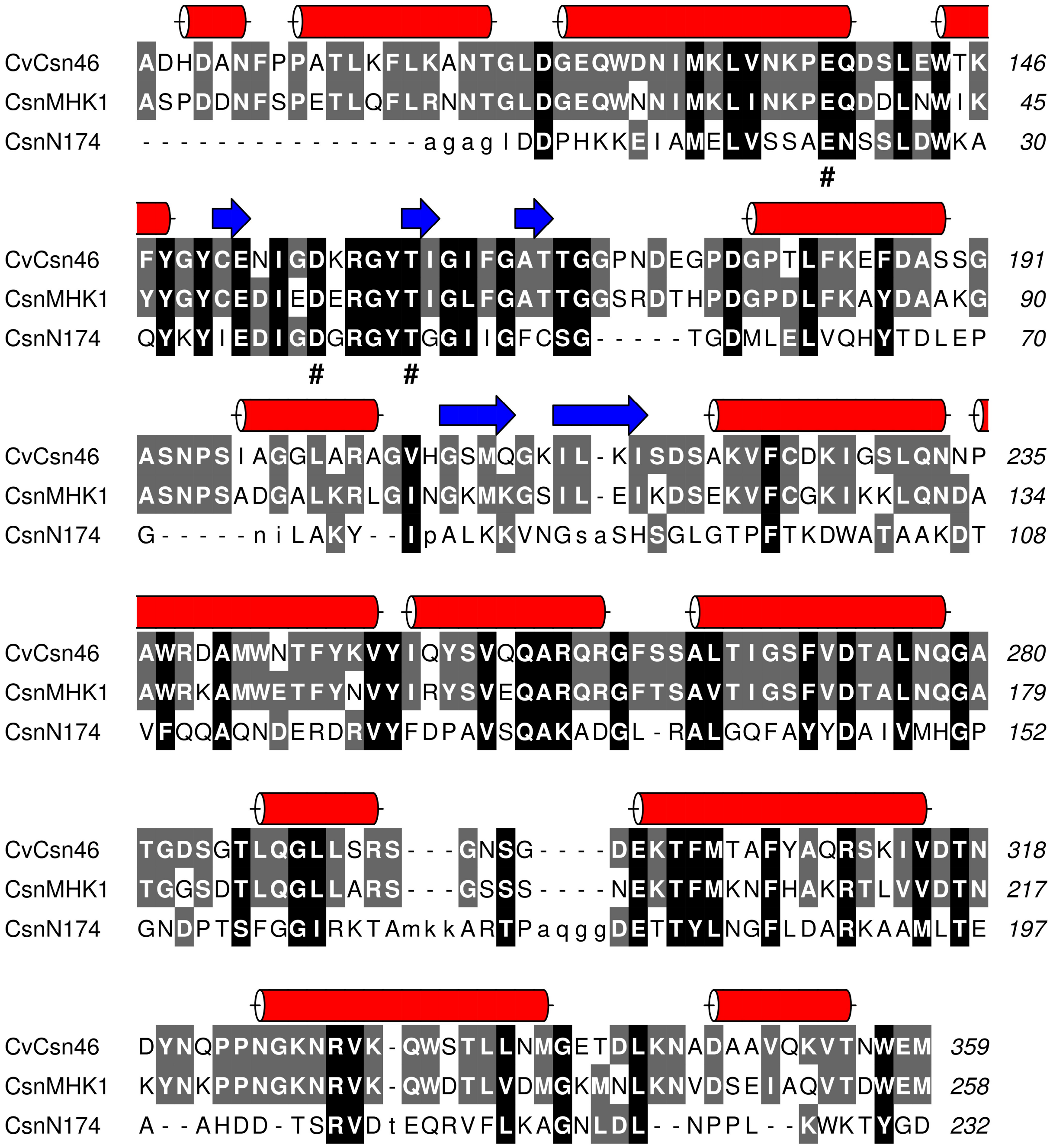


Figure S9. Multiple structural alignment of the GH46 CatDs of *Cv*Csn46, CsnMHK1 and CsnN174. The alignment was generated by submitting the 3D coordinates of the *Cv*Csn46 model structure (apo-form) to the DALI web server (http://ekhidna2.biocenter.helsinki.fi/dali) [6] and selecting among the matched structures those of CsnMHK1 from *N. circulans* (PDB ID: 1QGI; Z-core = 46.0, RMSD = 0.1 Å, 69% sequence identity) and CsnN174 from *Streptomyces* sp. N174 (PDB ID: 1CHK; Z score = 21.8, RMSD = 2.7 Å, 24% sequence identity) [7] to generate the multiple structural alignment. Uppercase and lowercase indicate structurally equivalent positions and insertions relative to *Cv*Csn46, respectively. Sites containing the catalytic residues are indicated by hash tags. The secondary structural elements of *Cv*Csn46, as assigned by the DSSP algorithm [8,9], are depicted as red cylinders and blue arrows. The alignment was edited using the program ALINE [10].

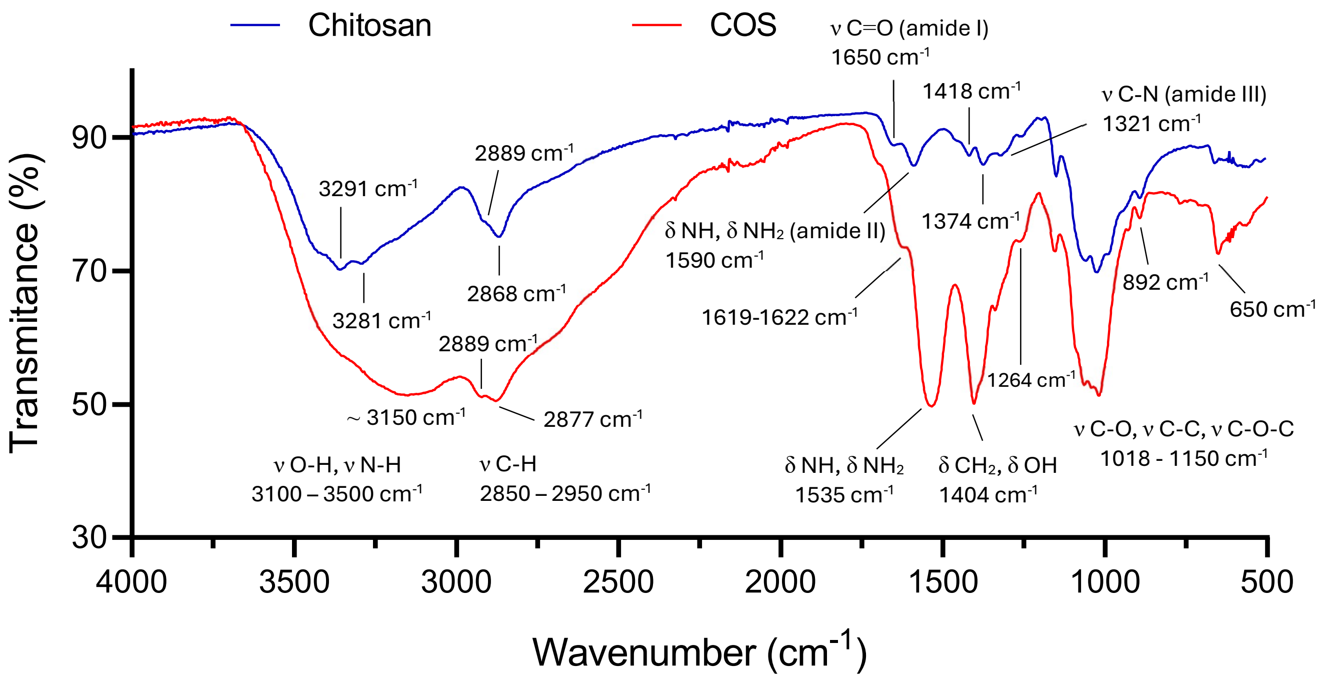


Figure S10. Fourier transform infrared (FTIR) spectroscopy analysis. FTIR spectra of original commercial chitosan and the chitooligosaccharides (COSs) released from colloidal chitosan incubated with *Cv*Csn46. The main absorption peaks (chitosan spectrum) were assigned as follows: O–H stretching vibrations overlapping with N–H stretching vibrations of the primary amine, as well as intra- and intermolecular hydrogen bonding of the polysaccharide (3100–3500 $cm^{-1}$); symmetric and asymmetric stretching vibrations of the C–H bond (2850–2950 $cm^{-1}$); N–H bending vibration of the primary amine (amide II; 1590 $cm^{-1}$); and symmetric and asymmetric stretching vibrations of the C1–O–C4 bridge and stretching vibrations of the pyranose ring C–C and C–O bonds (1000–1200 $cm^{-1}$). The bands at wavenumbers 1374 $cm^{-1}$ and 1418 $cm^{-1}$ were attributed to $CH_3$ symmetrical deformations and bending vibrations of $CH_2$ and the primary OH group (6-$CH_2$OH), respectively. The signals at approximately 1260 $cm^{-1}$ and 890 $cm^{-1}$ corresponded to bending vibrations of the OH groups (1264 $cm^{-1}$) and CH bending vibrations out of the plane of the pyranose ring (892 $cm^{-1}$). The low-intensity bands at 1650 $cm^{-1}$ (C=O stretching of amide I) and 1321 $cm^{-1}$ (C–N stretching of amide III) and the absence of a discernable peak at approximately 1550 $cm^{-1}$ (N–H bending of amide II), which was probably overlapped by other bands, indicated the residual presence of *N*-acetyl groups, confirming that the chitosan was not fully deacetylated. The COS spectrum was distinguished by two strong peaks at 1535 and 1404 $cm^{-1}$, which were assigned to the N–H bending vibrations of the primary amine (peak at 1535 $cm^{-1}$) and $CH_2$ bending and orientation of the primary hydroxyl group (peak at 1404 $cm^{-1}$). Other highly intense peaks in the COS spectrum were observed at 1264 $cm^{-1}$ (bending vibrations of OH), 892 $cm^{-1}$ (CH bending out of the plane of the pyranose ring) and

650 $cm^{-1}$ (out-of-plane bending of NH and OH groups and participation in hydrogen bonding). Another distinctive feature of the COS spectrum was the shift of some bands toward lower wavenumbers. For example, the peak ascribed to overlapping OH and NH stretching vibrations was broader and shifted from ~3285 $cm^{-1}$ to ~3150 $cm^{-1}$. Other shifts were observed in the bands attributed to $NH_2$ deformation (from 1590 $cm^{-1}$ to 1535 $cm^{-1}$) and $CH_2$ and primary OH bending vibrations (from 1418 $cm^{-1}$ to 1404 $cm^{-1}$).

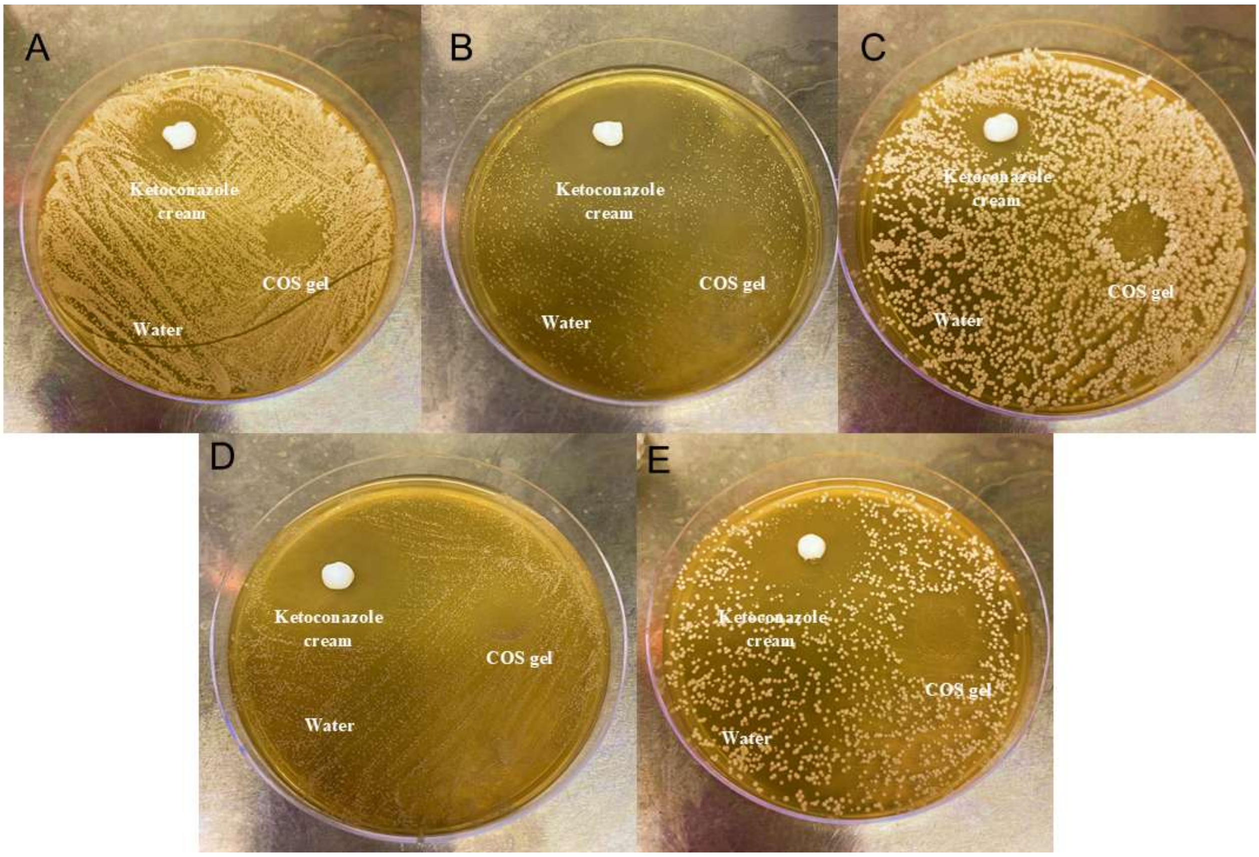


Figure S11. Antifungal activity of COS against non-*albicans Candida* strains. The antifungal effect was evaluated through a diffusion test on Sabouraud agar supplemented with droplets of 0.2% (m/v) agarose gel containing 0.5% (m/v) COS. The following *Candida* strains were used: *C. tropicalis* ATCC 13803 (A), *C. parapsilosis* ATCC 90018 (B), *C. krusei* ATCC 6258 (C), *C. parapsilosis* ATCC 22019 (D) and *C. tropicalis* ATCC 750 (E). A commercial ketoconazole-based antifungal cream (dose = 12 mg; CIMED Pharmaceutical Industry, Pouso Alegre, MG, Brazil) was used as a positive control.

Table S1. Diameters (mm) of growth inhibition zones of *Candida* strains cultivated on Sabouraud agar plates and exposed to droplets of 0.2% (m/v) agarose gel (dose = 15 mg) containing 0.5% (m/v) COS. A commercial antifungal cream containing 2% ketoconazole (dose = 12 mg) was included for comparison. Sterile distilled water (100 µL) was used as a negative control.

| **Strains** | **Agarose gel containing COS** | **Commercial antifungal cream** | **Water** |
|---|---|---|---|
| *Candida krusei* ATCC 6258 | 10 ± 1 | 10 ± 1 | 0 |
| *Candida parapsilosis* ATCC 22019 | 13 ± 2 | 25 ± 1 | 0 |
| *Candida parapsilosis* ATCC 90018 | 11 ± 1 | 28 ± 1 | 0 |
| *Candida tropicalis* ATCC 750 | 12 ± 1 | 14 ± 2 | 0 |
| *Candida tropicalis* ATCC 13803 | 24 ± 1 | 20 ± 1 | 0 |

The diameters of the growth inhibition zones were measured after incubation at 35 °C for 24 h. The values shown are the means ± SDs (n = 3 replicates).

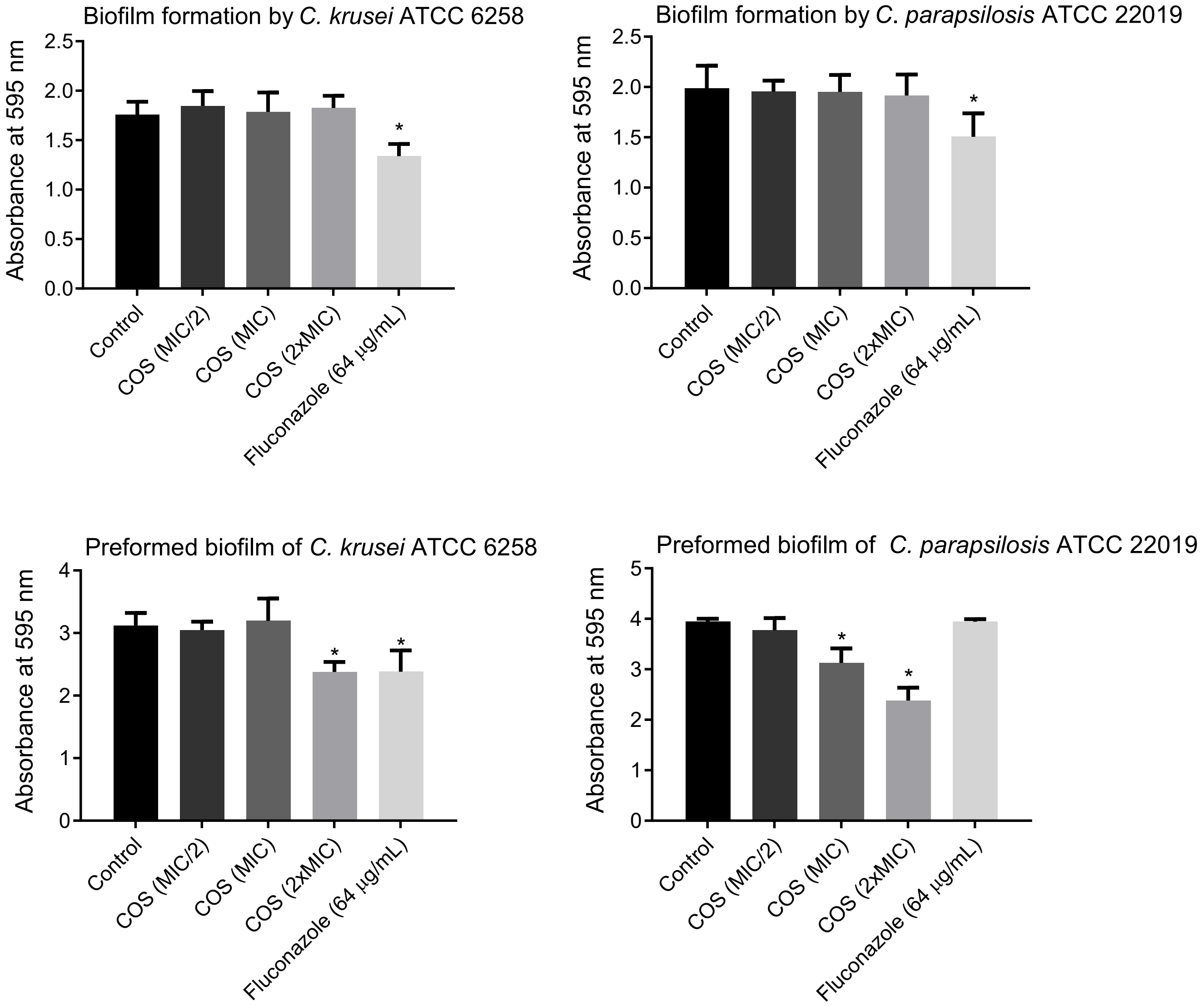


Figure S12. Antibiofilm activity of COS. The antibiofilm activity of COS was assessed via a crystal violet biofilm microtiter plate assay, as described in the methods section, subsection 2.8.4. The ability of COS to inhibit the formation of non-*albicans Candida* biofilms as well as its ability to disorganize a preformed biofilm was evaluated. The results are presented as the means ± SDs (n = 3 replicates). One-way ANOVA followed by Dunnett's test was used to evaluate statistical significance (*$p < 0.05$, compared with the control group).

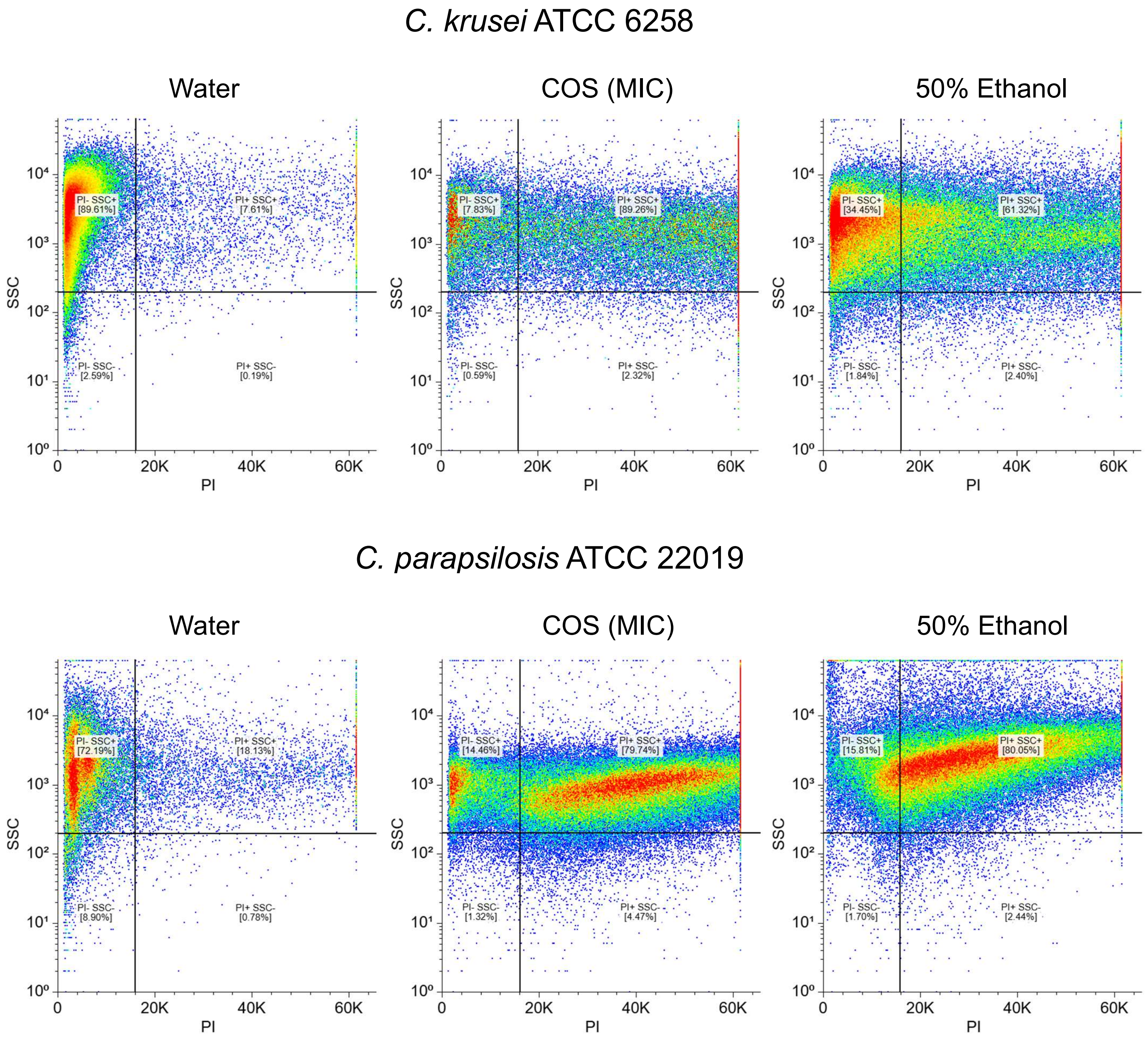


Figure S13. Density plots of propidium iodide (PI) fluorescence versus side scatter (SSC) of *Candida* cells after treatment with COS. The cells ($1 \times 10^6$ CFU/mL) were treated with COS (MIC = 312.5 μg/mL) for 1 h, centrifuged (5000 × *g* for 10 min at 4 °C), resuspended in 2 mL of 0.15 M NaCl, incubated with 1 μM PI for 10 min and analyzed via flow cytometry with a Partec GmbH flow cytometer (Münster, Germany). Cells treated with water or 50% (v/v) ethanol were used as negative and positive controls for cell death, respectively. Density plot graphics were generated via the Floreada.io server (https://floreada.io).

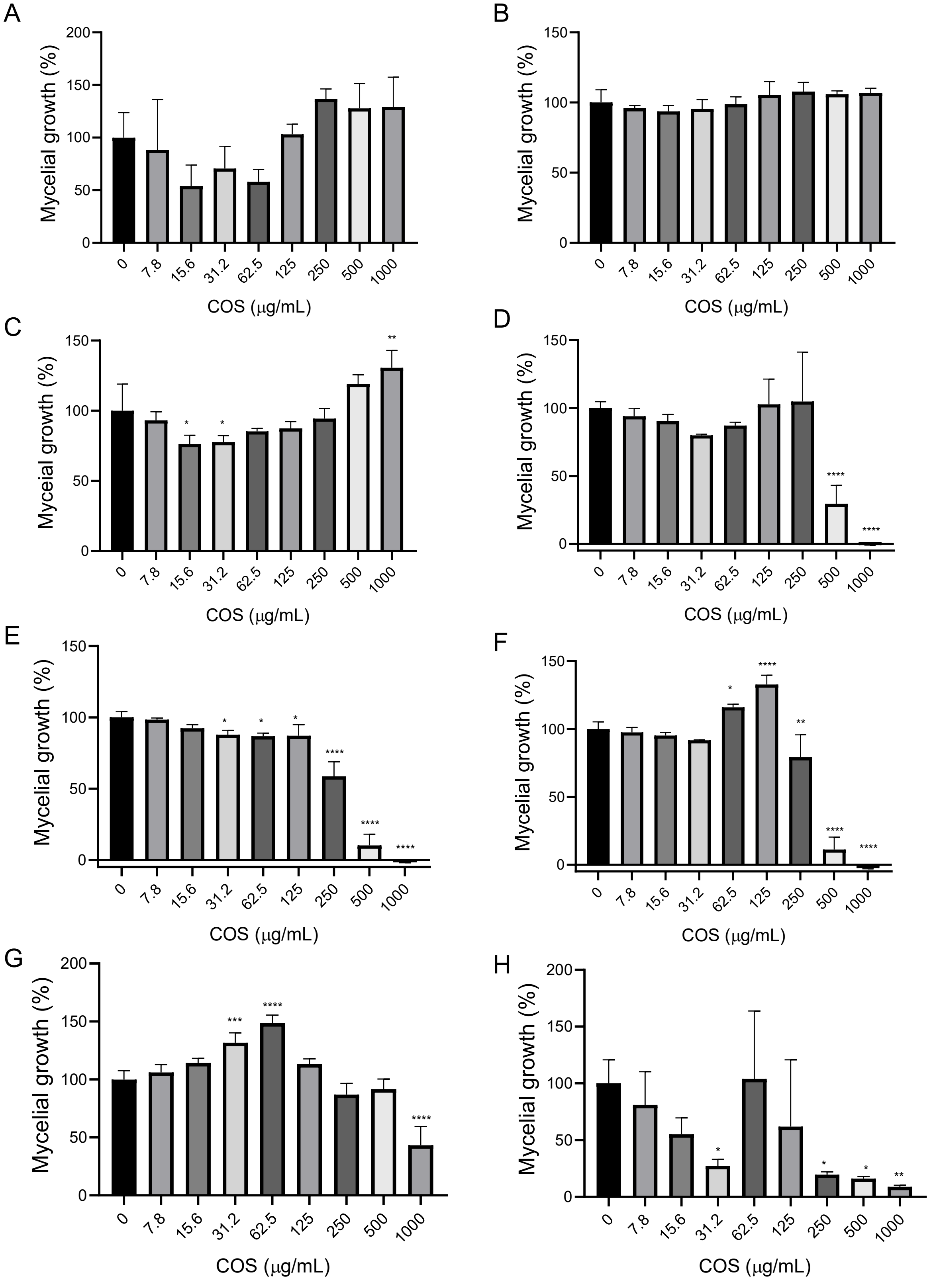


Figure S14. Effect of COS on the *in vitro* mycelial growth of *Colletotrichum gloeosporioides* (A), *C. lindemuthianum* (B), *C. scovillei* (C), *Fusarium lateritium* (D), *F. oxysporum* (E), *F. solani* (F), *Mucor circinelloides* (G) and *Penicillium decumbens* (H). The assays were performed as described in the methods section, subsection 2.11. The data are expressed as the means ± SDs (n = 3). One-way ANOVA followed by Dunnett's test was used to evaluate statistical significance (**p* < 0.05, ***p* < 0.01, ****p* < 0.001, *****p* < 0.0001).

## References


(1) Williams, C. J.; Headd, J. J.; Moriarty, N. W.; Prisant, M. G.; Videau, L. L.; Deis, L. N.; Verma, V.; Keedy, D. A.; Hintze, B. J.; Chen, V. B.; Jain, S.; Lewis, S. M.; Arendall, W. B.; Snoeyink, J.; Adams, P. D.; Lovell, S. C.; Richardson, J. S.; Richardson, D. C. MolProbity: More and Better Reference Data for Improved All-Atom Structure Validation. *Protein Sci.* **2018**, *27* (1), 293–315. https://doi.org/10.1002/pro.3330.

(2) Studer, G.; Rempfer, C.; Waterhouse, A. M.; Gumienny, R.; Haas, J.; Schwede, T. QMEANDisCo—Distance Constraints Applied on Model Quality Estimation. *Bioinformatics* **2020**, *36* (6), 1765–1771. https://doi.org/10.1093/bioinformatics/btz828.

(3) Waterhouse, A.; Bertoni, M.; Bienert, S.; Studer, G.; Tauriello, G.; Gumienny, R.; Heer, F. T.; de Beer, T. A. P.; Rempfer, C.; Bordoli, L.; Lepore, R.; Schwede, T. SWISS-MODEL: Homology Modelling of Protein Structures and Complexes. *Nucleic Acids Res.* **2018**, *46* (W1), W296–W303. https://doi.org/10.1093/nar/gky427.

(4) Saito, J.; Kita, A.; Higuchi, Y.; Nagata, Y.; Ando, A.; Miki, K. Crystal Structure of Chitosanase from Bacillus Circulans MH-K1 at 1.6-A Resolution and Its Substrate Recognition Mechanism. *J. Biol. Chem.* **1999**, *274* (43), 30818–30825. https://doi.org/10.1074/jbc.274.43.30818.

(5) Suzuki, M.; Saito, A.; Kobayashi, M.; Yokoyama, T.; Omiya, S.; Li, J.; Sugita, K.; Miki, K.; Saito, J.-I.; Ando, A. Crystal Structure of the GH-46 Subclass III Chitosanase from Bacillus Circulans MH-K1 in Complex with Chitotetraose. *Biochim. Biophys. Acta Gen. Subj.* **2024**, *1868* (3), 130549. https://doi.org/10.1016/j.bbagen.2023.130549.

(6) Holm, L.; Laiho, A.; Törönen, P.; Salgado, M. DALI Shines a Light on Remote Homologs: One Hundred Discoveries. *Protein Sci.* **2023**, *32* (1), e4519. https://doi.org/10.1002/pro.4519.

(7) Marcotte, E. M.; Monzingo, A. F.; Ernst, S. R.; Brzezinski, R.; Robertus, J. D. X-Ray Structure of an Anti-Fungal Chitosanase from Streptomyces N174. *Nat. Struct. Biol.* **1996**, *3* (2), 155–162. https://doi.org/10.1038/nsb0296-155.

(8) Joosten, R. P.; te Beek, T. A. H.; Krieger, E.; Hekkelman, M. L.; Hooft, R. W. W.; Schneider, R.; Sander, C.; Vriend, G. A Series of PDB Related Databases for Everyday Needs. *Nucleic Acids Res.* **2011**, *39* (suppl_1), D411–D419. https://doi.org/10.1093/nar/gkq1105.

(9) Kabsch, W.; Sander, C. Dictionary of Protein Secondary Structure: Pattern Recognition of Hydrogen-Bonded and Geometrical Features. *Biopolymers* **1983**, *22* (12), 2577–2637. https://doi.org/10.1002/bip.360221211.

(10) Bond, C. S.; Schüttelkopf, A. W. ALINE: A WYSIWYG Protein-Sequence Alignment Editor for Publication-Quality Alignments. *Acta Crystallogr. D Biol. Crystallogr.* **2009**, *65* (Pt 5), 510–512. https://doi.org/10.1107/S0907444909007835.
